# Modeling conformational transitions in DNA, RNA, and protein–nucleic acid complexes

**DOI:** 10.1093/nar/gkag807

**Published:** 2026-08-26

**Authors:** Domenico Scaramozzino, Marco Cannariato, Byung Ho Lee, Marco A Deriu, Laura Orellana

**Affiliations:** Protein Dynamics and Mutation Lab, Department of Oncology-Pathology, Karolinska Institute, Solna 17165, Sweden; PolitoBIOMed Lab, Department of Mechanical and Aerospace Engineering, Politecnico di Torino, Torino 10129, Italy; Protein Dynamics and Mutation Lab, Department of Oncology-Pathology, Karolinska Institute, Solna 17165, Sweden; PolitoBIOMed Lab, Department of Mechanical and Aerospace Engineering, Politecnico di Torino, Torino 10129, Italy; Protein Dynamics and Mutation Lab, Department of Oncology-Pathology, Karolinska Institute, Solna 17165, Sweden

## Abstract

The flexibility of nucleic acids plays a central role in numerous biological processes, including chromatin organization, gene regulation, and ribosome assembly. While elastic network models (ENMs) have successfully captured conformational changes in proteins through harmonic normal modes (NMs), analogous approaches for nucleic acids remain limited. Here, we introduce a generalized essential dynamics-refined ENM (edENM) for both DNA, RNA, and protein–nucleic acid complexes, parametrized against a diverse set of molecular dynamics simulations and validated using experimental ensembles from nuclear magnetic resonance, X-ray crystallography, and cryogenic electron microscopy. edENM achieves high agreement with experimental conformational changes across a curated benchmark of ∼60 DNA, RNA, and protein–nucleic acid systems. Compared to uniform-spring parametrizations, it produces significantly more collective NMs and suppresses unphysical backbone ruptures. We further integrate edENM into eBDIMS2, an efficient Brownian Dynamics path-sampling framework, extending its applicability to nucleic acid-containing systems at the megadalton scale. This enables the exploration of complex conformational transitions, including rearrangements of RNA folds in coronaviruses, large-scale remodeling in Argonaute–RNA complexes, multi-nucleosome assemblies in chromatin, as well as ribosomal particles. Together, these results establish an accessible and scalable elastic network framework for modeling conformational changes across the full spectrum of nucleic acid-containing biological systems.

## Graphical Abstract

**Graphical Abstract:**
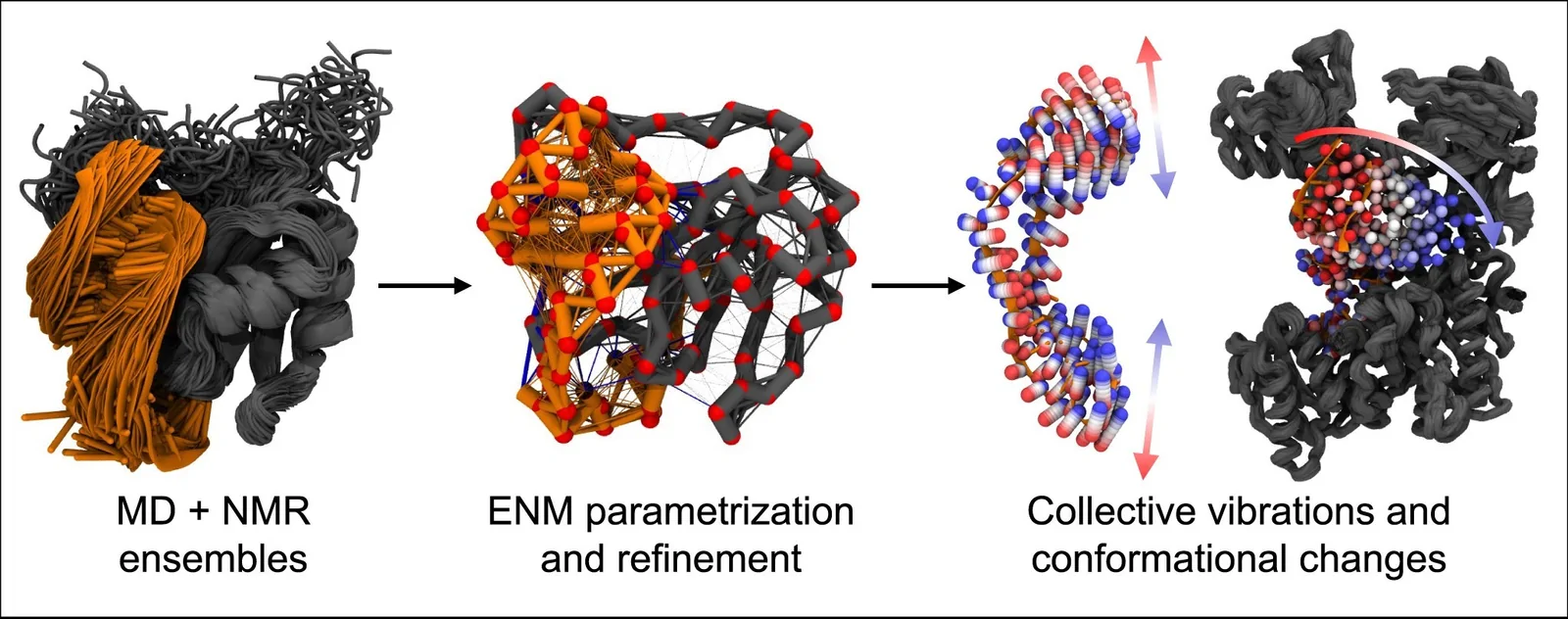

## Introduction

The conformational flexibility of DNA and RNA underlies many fundamental biological processes [1], including chromatin organization, gene regulation, RNA-guided target recognition, and ribosomal assembly [2–8]. These processes often involve coordinated structural rearrangements spanning large nucleic acid and protein–nucleic acid assemblies, from nucleosome remodeling [9] and ribosomal maturation [10] to RNA “slicing” [11]. The dynamics and flexibility of nucleic acids are deeply influenced by their interactions with proteins [12]. In protein–nucleic acid assemblies, local conformational changes can propagate through the entire complex, making a proper understanding of their collective dynamics essential to dissect biological function [12, 13].

Among the techniques that are employed to investigate the flexibility of biomacromolecules, the gold standard is molecular dynamics (MD) [14]. MD allows to simulate with atomistic details the dynamics of proteins and nucleic acids, while reproducing experimental measures and assisting in their interpretation [15–17]. MD simulations have provided pivotal insights into a variety of biological processes [14, 15]. Principal component analysis (PCA) of MD simulations, also referred to as essential dynamics (ED) [18], has also offered detailed insights into molecular flexibility [19, 20], capturing large-scale and biologically relevant motions [21]. Despite these advantages, MD simulations face major challenges, such as the significant computational demand and the so-called “sampling problem,” which limits the access to the long timescales and large amplitudes characteristic of functional conformational changes [22]. Several coarse-grained (CG) approaches have been developed to overcome the computational challenges posed by atomistic MD [23, 24]. In CG representations, the molecular structure is simplified in order to retain only the physical/chemical properties of major interest, reaching a compromise between computational efficiency and simulation accuracy.

Among CG methods, elastic network models (ENMs) describe the structure of macromolecules as a network of elastic springs in the harmonic regime [25–27]. These models have proven effective in describing the direction of biologically relevant motions, such as cooperative domain movements [20, 28–33]. Most ENM-based approaches characterize equilibrium fluctuations rather than continuous conformational changes between experimentally observed states—a limitation that is particularly important for nucleic acid assemblies, where functional motions frequently involve large-scale anharmonic rearrangements and coupled protein–nucleic acid remodeling. In proteins, ENMs often use a single node per amino acid localized at the position of the C_α_ atom [27], and the criteria to define the network connectivity and the spring constants differ significantly [34]. While a few ENMs have been proposed for nucleic acids, differing in the number and localization of CG nodes per nucleotide and in the criteria defining spring constants [35–39], existing parametrizations often exhibit unrealistic localized deformations (see below), require separate treatments for DNA and RNA, and lack a validated and unified framework for protein–nucleic acid complexes [40–42].

A step forward in the definition of an optimized ENM for proteins made use of extensive MD simulations as benchmark for the ENM parametrization instead of the traditionally used B-factors [29]. Atomic B-factors are strongly influenced by crystal packing and refinement errors [43, 44], while MD data capture both short- and long-timescale motions including anharmonicity effects, making them a more reliable parametrization target. The ED-based ENM (edENM) for proteins, refined on MD data and developed in Orellana *et al.* [29], was shown to outperform standard ENM approaches both regarding global and local flexibility and has been widely used [45, 46]. Further implementations of the edENM protein force field into a biased Brownian Dynamics (BD) simulation framework [47] resulted into the path-sampling algorithm eBDIMS [48], which allows to explore non-linear transition pathways between end-state conformations and generate reliable protein intermediates [49]. Recently, eBDIMS was substantially upgraded to eBDIMS2 [50], achieving quasi-linear scaling with system size and enabling the sampling of conformational transitions in megadalton protein assemblies previously inaccessible to other CG path-sampling methods.

Previous efforts to optimize ENM topologies for nucleic acids, using experimental structures, MD, and SHAPE experiments [36, 39], have been limited to either RNA or DNA separately, with no MD-based refinement attempted for nucleic acids and no extension to protein–nucleic acid complexes. Here, we introduce a general edENM, a unified ED-refined elastic network model for both nucleic acids (edENM-NA) and protein–nucleic acid complexes (edENM-PNA), parametrized against a diverse set of MD simulations and validated on a curated benchmark of ∼60 nuclear magnetic resonance (NMR), X-ray, and cryo-EM structural ensembles from the Protein Data Bank (PDB) [51]. Compared to uniform-spring ENMs, edENMs produce significantly more collective normal modes (NMs) and suppress unphysical backbone ruptures. We further integrate edENM-PNA into eBDIMS2 [49], extending its capabilities from megadalton protein assemblies to any nucleic acid–containing complex. The resulting approach enables the exploration of large-scale and anharmonic conformational changes in heterogeneous nucleic acid assemblies. Applications to coronavirus RNA stem-loops, Argonaute-RNA “slicing” states, multi-nuclosomes, and ribosomal particles demonstrate the ability of our framework to model a wide variety of biologically relevant conformational transitions across scales from ∼40 kDa isolated RNA folds to ∼1.3 MDa ribonucleoprotein assemblies.

## Materials and methods

### Parametrization workflow

**Figure 1.**
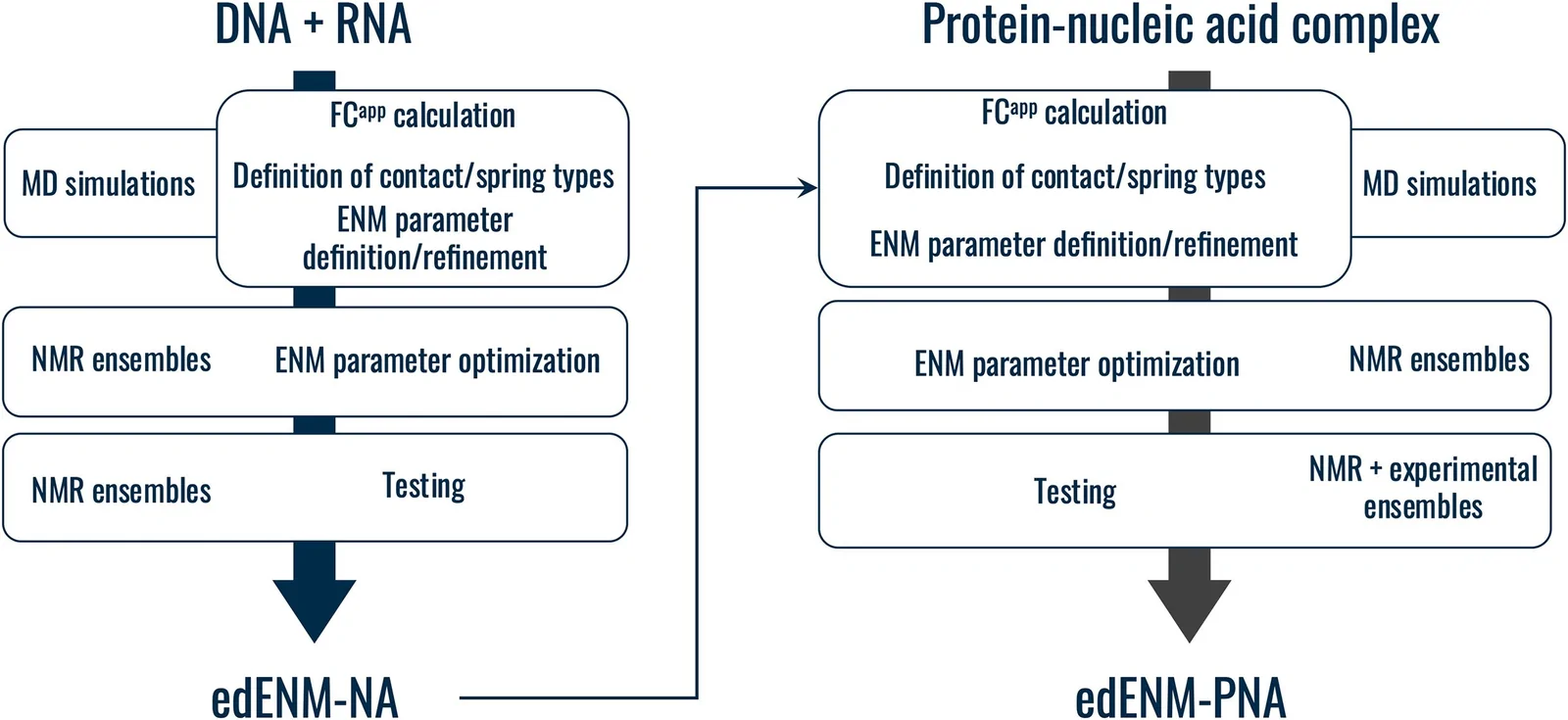
Workflow for the parametrization of: (left) edENM-NA for DNA and RNA; (right) edENM-PNA for protein–nucleic acid complexes. For both edENMs, the parametrization begins with the calculation of apparent force constants, FC^app^, for selected pairs of atoms (P, C1′, C2, C_α_) from the atomistic simulations. Contact types are then defined based on FC^app^ values depending on inter-bead distances, followed by an initial definition of the ENM parameters (spring constant *C*, distance-dependent decay parameter *n*, and cutoff for long-range interactions) and further refinement based on FC^app^ data. A second refinement step optimizes the network parameters to maximize the similarity between the ENM NMs and the principal components (PCs) from NMR ensembles. Finally, the performances of both models are assessed by comparing NMs with the PC vector directions obtained from additional NMR ensembles and, in the case of protein–nucleic acid complexes, from X-ray/cryo-EM structural ensembles.

The parametrization of the new edENM (Fig. 1) has been performed in two phases: (i) parametrization of the ENM for DNA and RNA (edENM-NA); (ii) definition of the interface contacts between the nucleic acid and the protein, and integration of edENM-NA with the protein edENM developed in Orellana *et al.* [29], resulting in edENM-PNA for protein–nucleic acid complexes. For the edENM-NA topology, we used the three-bead sugar-base-phosphate (SBP) model proposed by Pinamonti *et al.* [39] (Supplementary Fig. S1). We chose the SBP topology as it was considered to be a good compromise between network complexity and model accuracy [39]. In the original SBP model, all springs have the same force constant and a spatial interaction cutoff of 9 Å, and each nucleotide group is represented by a single bead, C1′ representing the sugar, C2 the nitrogenous base, and P the phosphate group (Supplementary Fig. S1) [39].

After defining the initial SBP topology, we used an MD-based parametrization coupled with further refinement based on experimental data to develop edENM-NA and edENM-PNA. In the first phase of the parametrization (edENM-NA), we estimated the apparent force constants between P, C1′, and C2 atoms from a training set of nucleic acid MD trajectories (see below) as [29, 52]:


(1) \begin{eqnarray*}
\mathrm{ FC}_{ij}^{\mathrm{ app}} = \frac{{{{k}_B}T}}{{\left\langle {{{{\left( {{{d}_{ij}} - d_{ij}^a} \right)}}^2}} \right\rangle }},
\end{eqnarray*}

where *d_ij_* represents the instantaneous distance between atoms *i* and *j, d_ij_^a^* the average value of this distance along the trajectory, *k_B_* the Boltzmann constant, *T* is the temperature, and ⟨…⟩ stands for the averaging across all MD frames. To obtain a first description of the edENM-NA spring constants, the values ofFC*_ij_*^app^ were mapped against the corresponding average distances *d_ij_^a^*, and the FC*_ij_*^app^ – *d_ij_^a^* relationships were used to define different contact (spring) types. When a distance-dependent relationship was observed, this was fitted using an inverse exponential function as in Orellana *et al.* [29]:


(2) \begin{eqnarray*}
{{k}_{ij}} = {{\left( {\frac{C}{{d_{ij}^a}}} \right)}^n},
\end{eqnarray*}

where *k_ij_* represents the edENM-NA spring force constant, and *C* and *n* are the parameters associated with the strength and the distance-dependent decay of the spring, respectively. A first estimation of these parameters was performed by minimizing the standard error of the *k_ij_* regression curve against FC*_ij_*^app^–*d_ij_^a^* MD values. These parameters were then refined performing a random search in the neighborhood of the estimated variables, using a random combination of 1500 values. Optimal values of *C* and *n* were subsequently found by comparing the vector alignment (overlap) between the NMs from normal mode analysis (NMA) of the edENM-NA and the ED eigenvectors from each MD trajectory (see below). After this refinement stage, the best parameters that maximize root mean square inner products (RMSIPs, see below) were compared using different cutoff values, ranging from 9 to 14 Å. In this step, the NMs were also compared against PCs derived from experimental ensembles of DNA and RNA molecules obtained from multi-model PDB files from solution NMR. Once the optimal parameters were selected, we tested the resulting edENM-NA against the remaining MD simulations and an additional dataset of NMR and multi-state ensembles (see below). The performance of the resulting edENM was compared against the uniform-spring SBP-ENM from Pinamonti *et al.* [39].

In the second parametrization phase, we estimated the apparent force constants FC*_ij_*^app^ for protein–nucleic acid interface contacts from an additional set of training MD simulations of protein–nucleic acid complexes. A first definition and refinement of spring constants was performed making using of the inverse exponential function from equation (2). A random search in the neighborhood of the *C* and *n* parameters was performed using a combination of 1100 values and considering several interaction cutoffs. The parameters of the edENM-PNA spring constants and the cutoff for protein–nucleic acids contacts were then refined using the training MD trajectories and performing an additional optimization based on experimental ensembles from NMR. Finally, the performance of the model was tested considering a separate set of MD trajectories, the NMR dataset, as well as a dataset of structural ensembles from X-ray and cryo-EM experiments that characterize conformational changes in protein–nucleic acid complexes (see below).

### Dataset of molecular dynamics simulations

To perform an MD-based parametrization, we retrieved several MD trajectories from the existing literature using *MDverse* [53], and making use of *Zenodo, OSF*, and *figshare* online repositories. Molecular systems composed of DNA, RNA, as well as protein–nucleic acid complexes were considered. In total, we retrieved separate MD simulations for 11 DNA, 5 RNA, and 10 protein–nucleic acid systems. These systems were simulated using different high-performance simulation software, such as GROMACS [54], OpenMM [55], NAMD [56], AMBER [57], and DESMOND [58], as well as a variety of atomistic force fields, including AMBER03 [59], AMBER99 [60], AMBERff14SB [61], AMBERff19SB [62], CHARMM36 [63–65, 66], Drude-2017 [67–69], and OPLS4 [70], coupled with several refined parametrizations for DNA [71–73] and RNA [74, 75]. For each system, the number of available replicas ranged between 1 and 20, with simulation lengths ranging from a minimum of 30 ns to a maximum of 2 μs. Aggregating trajectories related to different molecular systems, simulated under diverse conditions and using different potentials, is important to minimize system- and force-field-dependent biases and increase the generalization of the elastic network parametrization. Detailed information about the MD dataset is reported in Supplementary Table S1.

For nucleic acids, 12 simulations were included for training and 4 for testing, while 7 simulations of protein–nucleic acid complexes were included for training and the remaining 3 for testing. For each simulation, we discarded the first 10 ns as these generally include initial equilibrations. Large-scale and anharmonic conformational changes might arise in longer simulations, and these motions are generally difficult to capture with NMs from ENMs due to their harmonic and small-amplitude nature. For this reason, we split each MD trajectory in smaller windows. For total simulation lengths <1 μs, we extracted MD windows of 50 ns, while for longer simulations we used windows of 100 ns. Since some local conformational changes might end up being split into adjacent windows, we also considered an overlap of 10 ns between adjacent windows. In cases where the total simulation length was <50 ns, the whole trajectory was taken as a single window.

On the resulting “windowed” trajectories, we performed PCA. PCA is a statistical technique used to capture the low-dimensional variables that explain the majority of the variance within high-dimensional data. PCA of MD simulations is usually referred to as ED and was shown to provide meaningful insights into protein dynamics and flexibilities [18, 20]. The input of PCA is an *c* × 3*N* coordinate matrix, ***X***, where *c* is the number of conformations in the MD trajectory and *N* the number of atoms-residues, usually considering only C_α_ atoms for proteins and P atoms for nucleic acids. From ***X***, the elements of the symmetric covariance matrix, ***C***, can be calculated as:


(3) \begin{eqnarray*}
{{c}_{ij}} = \left\langle {\left( {{{x}_i} - \left\langle {{{x}_i}} \right\rangle } \right)\left( {{{x}_j} - \left\langle {{{x}_j}} \right\rangle } \right)} \right\rangle ,
\end{eqnarray*}

where the brackets ⟨…⟩ indicate the average over all MD simulation frames. Eigenvalue–eigenvector decomposition is then used to diagonalize the covariance matrix as:


(4) \begin{eqnarray*}
{\boldsymbol{C}} = {\boldsymbol{U\Delta }}{{{\boldsymbol{U}}}^T},
\end{eqnarray*}

where the diagonal matrix ***Δ*** contains the eigenvalues of ***C***, while the matrix ***U*** contains its eigenvectors, representing the PCs. Eigenvalues are sorted in descending order and are directly proportional to the amount of variance explained by the corresponding PC eigenvector. Each PC eigenvector can be directly compared to NMs via overlaps and RMSIP scores (see below).

### Dataset of experimental ensembles and conformational changes

To refine the model parameters so that the dynamics of the edENM-NA and edENM-PNA also captures experimentally observed conformational changes, we made use of experimentally available nucleic acid structures and protein–nucleic acid complexes from the PDB [51]. For systems composed of solely nucleic acids (DNA or RNA), we retrieved a total of 29 ensembles, 25 of which are single NMR models, 3 are ensembles of multiple NMRs or multi-model cryo-EM structures, and one is an ensemble of distinct models from cryo-EM. Out of these 29 ensembles, 10 were used for parameter refining and 19 for testing. The set of all PDB models is listed in Supplementary Table S2.

We also retrieved experimental structures of protein–nucleic acid complexes from NMR models. After removing duplicates and NMRs with only one single model uploaded, we retrieved a list of 208 potentially usable NMR structures. Then we inspected these models and retained only those with a relevant conformational diversity. This was enforced by computing root mean square fluctuations (RMSFs) and root mean square deviations (RMSDs). First, we retained only those protein–nucleic acid complexes where maximum RMSD values between any two NMR models were >3 Å. Then, we removed portions of the structure where RMSFs were localized at the N- and C-termini of protein chains, which is common in NMR ensembles. This was done since these types of localized conformational changes at the protein tails are not the typical targets of ENM analyses, which tend to provide meaningful information on more collective motions. Finally, we re-computed RMSD values on the models without the flexible tails and retained only those with a global RMSD still >3 Å. These RMSD- and RMSF-based filters led us to retain a total of 21 significant protein–nucleic acid NMRs (Supplementary Table S3), that were used for edENM-PNA parameter refinement and testing.

In the case of ensembles and conformational changes in protein–nucleic acid complexes coming from sets of different X-ray and cryo-EM structures, we carried out a search of structural ensembles by performing a screening of the PDB and UniProt database [76]. First, all proteins with solved 3D structures and reviewed UniProt entries were considered (35 348 entries, as of May 2024). Then, we retained only those PDBs where at least one protein entity and one nucleic acid entity were present. UniProt IDs having only one associated PDB were discarded, reducing the suitable entries to 4258. UniProt IDs sharing the same PDBs, e.g. due also to the presence of heteromeric protein–protein complexes, were merged into the same UniProt-PDB combination, which led us to 2374 unique groups of PDB models. Then, entries with one or more proteins having a molecular weight higher than 400 kDa were filtered out in order not to consider gigantic protein–nucleic acid assemblies for harmonic NM testing. Then, we computed RMSDs between all PDBs via US-Align [77], maintaining only those with RMSDs >3 Å in order to ignore ensembles with negligible conformational diversity. Finally, we filtered out structures with low-resolution (>5 Å) and removed assemblies including >10 different polymer entities, which led to a final list of 265 potential ensembles/conformational changes. This final list of ensembles was then manually inspected to exclude inadequate cases due to, e.g. nucleic acids with sequences not matching in the different conformers, ensembles resulting in disconnected regions due to many missing residues, high RMSDs solely due to the motion of terminal tails, etc. Eventually, the final testing set was composed of 26 structural ensembles with at least 2 distinct PDB models that satisfied all our filtering criteria. The full list of ensembles with the included PDB models is reported in Supplementary Table S4. For each set of testing NMR models and conformational ensemble, we carried out PCA and extracted PCs. PC eigenvectors were then compared with NMs from edENM through overlap and RMSIP scores (see below).

### Normal mode analysis and comparison metrics for edENM optimization and testing

NMA is a mathematical technique used to extract the vibrational features of a structure. In the past decades, NMA has been widely applied to investigate the dynamics of proteins, nucleic acids, and biomacromolecules in general [25, 78]. NMA relies on the definition of the Hessian matrix, which contains the second derivatives of the potential energy of the system. Atomistic NMA often requires a preliminary minimization of the system and employs MD-based force fields accounting for energy variations in bond lengths, bond angles, dihedral angles, and long-range interactions [78]. On the contrary, ENM-NMA relies on a simpler harmonic potential that treats the structure as a network of elastic springs and allows to avoid the minimization step as it relies on the assumption that the ENM conformation is already in the energy minimum [25, 27]. To compute NMs for comparison with ED eigenvectors from MD trajectories, the ENM was built starting from the coordinates of the MD frame closest to the average conformation within each MD window. Experimental PDBs were instead used directly as reference topologies when comparing NMs to experimental PCs.

Once the molecule topology is determined and spring parameters are established, the 3*N* × 3*N* Hessian matrix ***H*** is assembled as a combination of 3 × 3 symmetric sub-blocks ***H_ij_*** [79]. Each sub-block contains the stiffness information about the interaction between nodes *i* and *j*:


(5) \begin{eqnarray*}
&&{{{\boldsymbol{H}}}_{ij}} = - \frac{{{{k}_{ij}}}}{{{{d}_{ij}}^2}}\\&& \left[ {\begin{array}{@{}*{3}{c}@{}} {{{{\left( {{{x}_j} - {{x}_i}} \right)}}^2}}&{\left( {{{x}_j} - {{x}_i}} \right)\left( {{{y}_j} - {{y}_i}} \right)}&{\left( {{{x}_j} - {{x}_i}} \right)\left( {{{z}_j} - {{z}_i}} \right)}\\ {\left( {{{x}_j} - {{x}_i}} \right)\left( {{{y}_j} - {{y}_i}} \right)}&{{{{\left( {{{y}_j} - {{y}_i}} \right)}}^2}}&{\left( {{{y}_j} - {{y}_i}} \right)\left( {{{z}_j} - {{z}_i}} \right)}\\ {\left( {{{x}_j} - {{x}_i}} \right)\left( {{{z}_j} - {{z}_i}} \right)}&{\left( {{{y}_j} - {{y}_i}} \right)\left( {{{z}_j} - {{z}_i}} \right)}&{{{{\left( {{{z}_j} - {{z}_i}} \right)}}^2}} \end{array}} \right]\\ ,
\end{eqnarray*}

where *k_ij_* is the stiffness of spring in the network connecting nodes *i* and *j, d_ij_* their distance, and *x, y, z* are the 3D coordinates derived from the X-ray, NMR, cryo-EM model, or the MD simulation frame. Once the Hessian matrix is assembled, its eigenvalues and eigenvectors are computed based on a standard matrix diagonalization, as already reported in equation (4). In this case, the diagonal matrix ***Δ*** contains the eigenvalues of ***H***, ordered by ascending value, and the column of ***U*** provide the full set of 3*N* eigenvectors. Each eigenvalue–eigenvector pair is associated with a specific NM of vibration: the eigenvalues describe the NM vibrational frequencies and are also associated to the NM energy and amplitude (low-frequency NMs are also the lowest-energy and generally the most collective), while eigenvectors describe the 3D directions along which NMs take place. The first six NMs describe zero-frequency motions associated with global translations and rotations and are discarded from following analysis.

The effectiveness of the method in capturing conformational changes was assessed by comparing the directions of NMs to: (i) the ED vectors from MD, (ii) the PCs from NMR and multi-model PDB structures, and (iii) the PCs from a collection of different PDB structures within a common experimental ensemble. For this purpose, we computed overlaps between NMs and PCs. The overlap *O_ij_* between a NM eigenvector ***ν****_i_* and an ensemble PC eigenvector ***w****_j_* is computed according to the following equation:


(6) \begin{eqnarray*}
{{O}_{ij}} = \frac{{\left| {{{{\boldsymbol{\nu }}}_i} \cdot {{{\boldsymbol{w}}}_j}} \right|}}{{\|{{{\boldsymbol{\nu }}}_i}\|\ \|{{{\boldsymbol{w}}}_j}\|}},
\end{eqnarray*}

where ‖***u***‖ indicates the Euclidean norm of the vector ***u***. An overlap of 0 implies that the two vectors are orthogonal, while for two matching (parallel) vectors the overlap is 1. Based on the first *n* NM and PC vectors, we also evaluated the maximum pairwise overlap, *O*_max_, defined as


(7) \begin{eqnarray*}
{{O}_{\mathrm{ max}}} = \mathop {\max }\limits_{i,j \le n} {{O}_{ij}}.
\end{eqnarray*}

On the other hand, the RMSIP expresses the similarity between two vectorial sub-spaces described by *n* vectors ***ν*** and ***w***, and it was computed as


(8) \begin{eqnarray*}
\mathrm{RMSI}{{\mathrm{ P}}_n} = \sqrt {\frac{1}{n}\mathop \sum \limits_{i = 1}^n \mathop \sum \limits_{j = 1}^n {{O}_{ij}}^2} .
\end{eqnarray*}

Typically, RMSIP calculations are restricted to the first NMs, e.g. *n* = 3, 5, or 10 modes, as the lowest-frequency motions typically represent functional motions [28, 33, 80]. Another useful metric to assess the global versus localized nature of conformational motions from PC or NM eigenvectors is the collectivity degree *κ*, which is defined as [28]


(9) \begin{eqnarray*}
\kappa = \frac{1}{N}\exp \left( { - \mathop \sum \limits_{i = 1}^N \frac{{{{\delta }_i}}}{{\mathop \sum \nolimits_{i = 1}^N {{\delta }_i}}}\ln \frac{{{{\delta }_i}}}{{\mathop \sum \nolimits_{i = 1}^N {{\delta }_i}}}} \right),
\end{eqnarray*}

where *δ_i_* stands for the absolute value of the displacement in the PC or NM eigenvector at residue *i*. The collectivity degree varies from a minimum of 1/*N*, if only one residue participates to the motion, to a maximum of 1, if all residues are uniformly involved in the structural change. The former case is associated with extremely localized motions, the latter with highly collective vibrations.

### Implementation in eBDIMS for path-sampling of large conformational transitions

After assessing the performance of the edENM-PNA, we integrated this new force-field into the eBDIMS [48, 49]. eBDIMS is a CG path-sampling algorithm that relies on a protein ENM representation and a BD simulation framework, and can be used to track intermediate states along transition pathways by using a Dynamic IMportant Sampling (DIMS) bias [49]. eBDIMS was shown to produce realistic protein intermediates along transition pathways and capture experimentally observed intermediates. eBDIMS was also recently upgraded to a more efficient version, eBDIMS2, that can now simulate large-scale and complex transitions in gigantic protein assemblies from cryo-EM [50]. We implemented the new edENM parameters for nucleic acids and protein–nucleic acid contacts into eBDIMS2, so that we can now simulate large-scale conformational transitions not only in proteins, but also in DNA, RNA, as well as a wide variety of protein–nucleic acid systems (eBDIMS2-PNA), with virtually no restriction on system size, macromolecular architecture, and motion complexity. Here, we applied eBDIMS2-PNA to simulate conformational changes in four different systems: a classical hinge-bending motion in the *BtCoV-HKU5* 5′ proximal stem-loop 5 (SL5) RNA (∼40 kDa), a localized RNA transition from the central-duplex to the bent-duplex conformation of *Arabidopsis thaliana* Argonaute 10 (∼120 kDa), a large-scale opening of an *Homo sapiens* telomeric tri-nucleosome (∼560 kDa), as well as a hinge-bending motion in the large 18S ribosomal RNA (rRNA) of an *Homo sapiens* pre-40S ribosomal subunit (∼1.3 MDa).

## Results

### Parametrization and testing of edENM-NA

**Figure 2.**
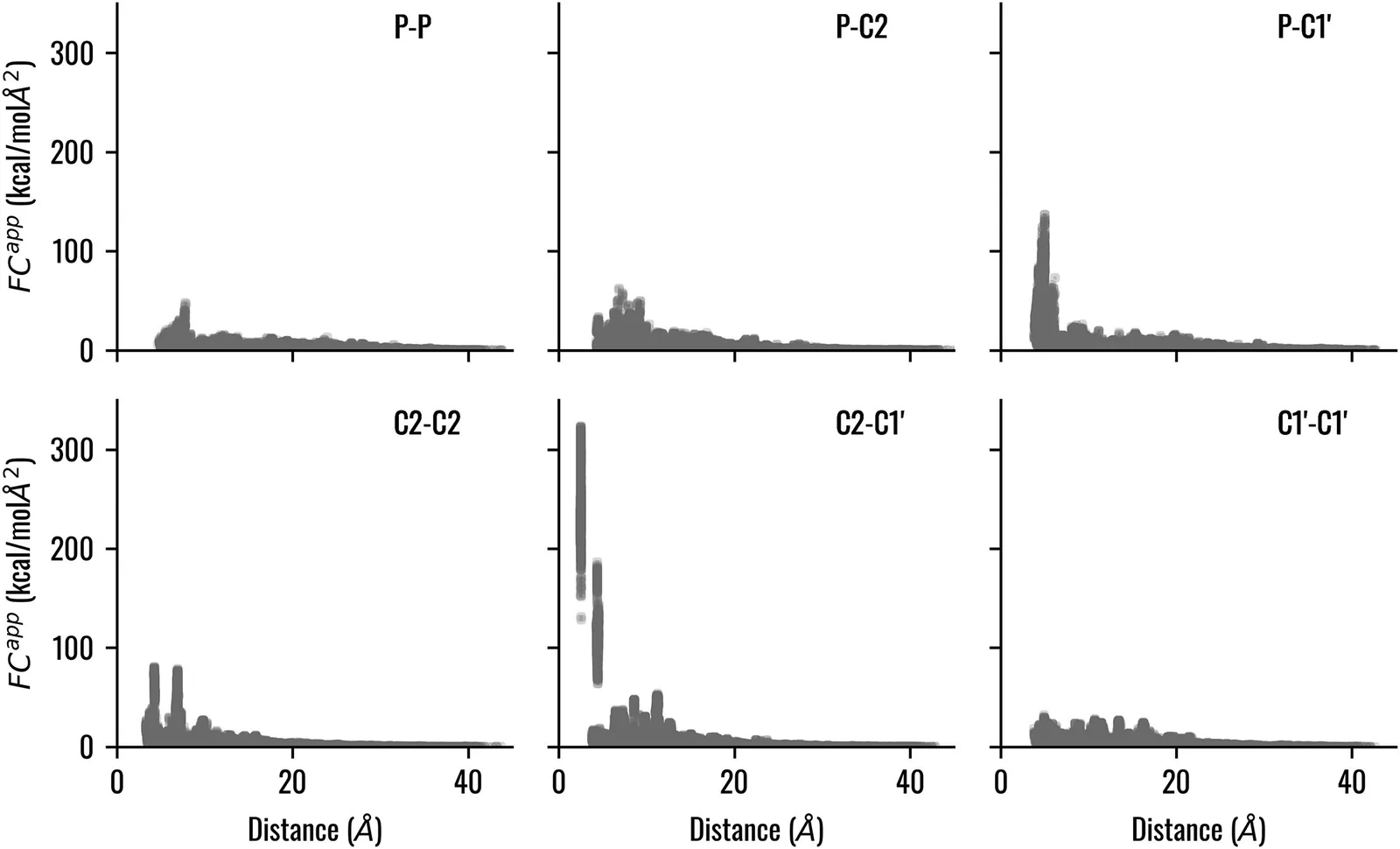
Distribution of the apparent force constants FC*_ij_*^app^ as a function of the average distance *d_ij_^a^* in the training MD dataset of nucleic acids systems, aggregating both DNA and RNA systems. The apparent force constants between different interacting couples considering P, C1′, and C2 beads are shown.

**Figure 3.**
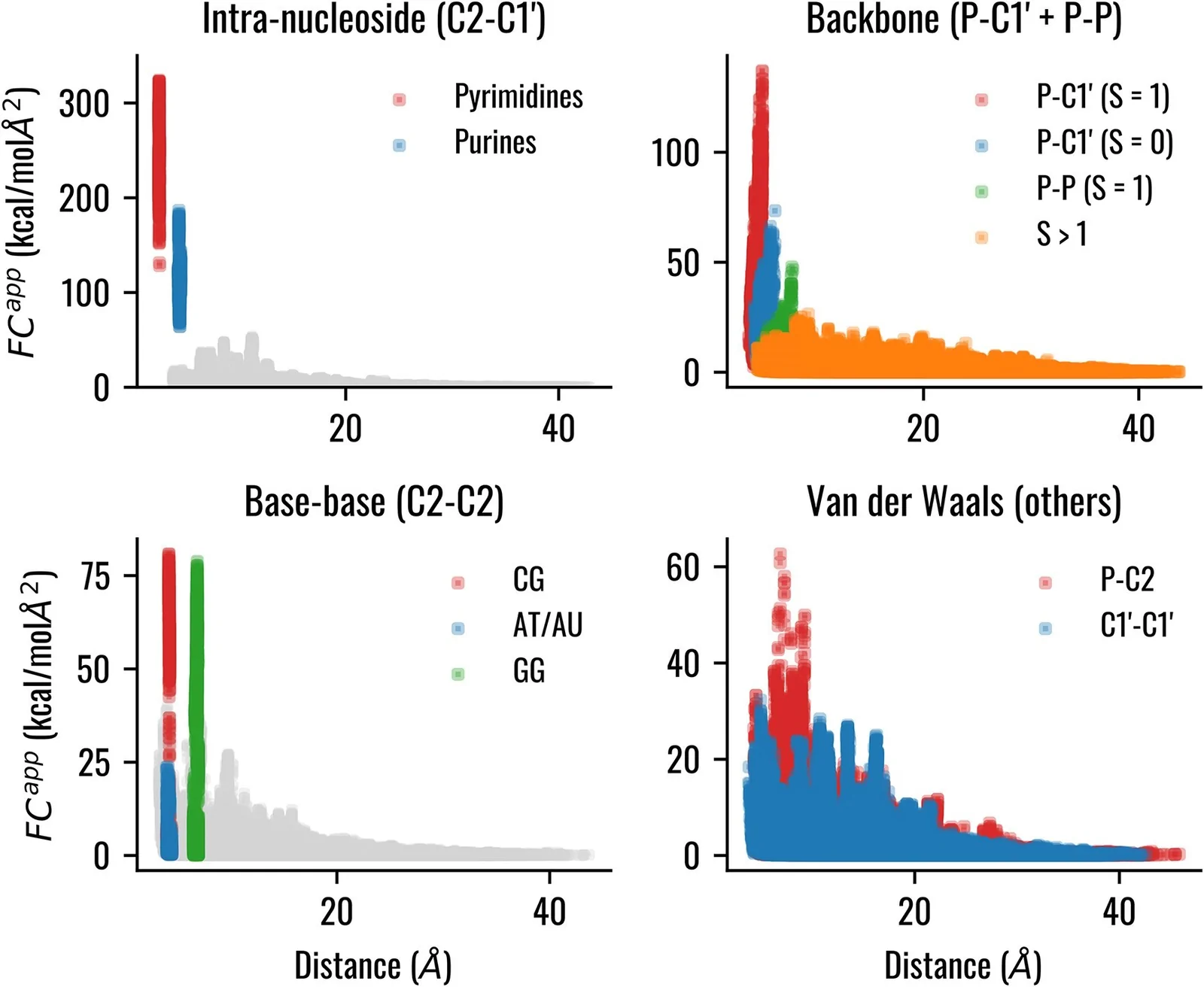
Definition of four contact classes used in the edENM-NA, identified from the distribution of apparent force constants FC*_ij_*^app^ as a function of the average inter-bead distance *d_ij_^a^* from MD simulations. Upper left: “intra-nucleoside” contacts between C2 and C1′ beads within the same nucleotide (*S* = 0), describing the rigidity of the sugar-base moiety. Red and blue points correspond to pyrimidines and purines, respectively. Gray points represent C2–C1′ contacts between different nucleotides (*S* > 0), which are aggregated to “Van der Waals” contact class. Upper right: “backbone” contacts between P and C1′ beads, defining the CG nucleic acid backbone. Red points correspond to P–C1′ contacts between neighboring nucleotides (*S* = 1), blue points to P–C1′ contacts within the same nucleotide (*S* = 0), green points to adjacent P–P contacts (*S* = 1), and orange points to all P–C1′ and P–P contacts between nucleotides farther apart along the sequence (*S* > 1). Lower left: “base–base” contacts between C2 beads. Red and blue points correspond to canonical CG and AT/AU base pairs, respectively, with the stronger CG interactions reflecting the additional hydrogen bond in CG pairs compared to AT/AU. Green points identify the longer-range GG stacking contacts observed in a DNA β-hairpin, which exhibit FC*_ij_*^app^ values comparable to those of CG base pairs. Gray points represent all remaining C2–C2, which are grouped in the “Van der Waals” contact class. Lower right: “Van der Waals” contacts, comprising all remaining non-bonded bead pairs (P–C2 in red, C1′–C1′ in blue), which are modeled as long-range interactions with distance-dependent force constants.

The MD-based ENM parametrization begins with the calculation of the apparent force constants (FC*_ij_*^app^, equation 1) from the dataset of training MD trajectories (Supplementary Table S1). The distributions ofFC*_ij_*^app^ values, which describe the interaction strength between pairs of atoms throughout the MD trajectories, were analyzed as a function of the interatomic distances for the three beads in our edENM-NA model (P, C1′, C2). Putative differences between DNA and RNA systems were investigated (Supplementary Figs S2 and S3). However, comparing individual distributions of FC*_ij_*^app^ values with those obtained aggregating DNA and RNA trajectories (Fig. 2), did not reveal major differences in the four main types of contacts identified (see below) to justify distinct parametrizations between the two nucleic acids at the CG level. Therefore, for the sake of simplicity, a general edENM-NA force-field was parametrized for both DNA and RNA systems. The overall distributions of FC*_ij_^app^* values in the training trajectories are shown in Fig. 2, which made us define four interaction types considering interactions between P, C1′, and C2 beads (Fig. 3).

The first class comprises “intra-nucleoside” contacts, defined between the C2 and C1′ beads within the same nucleotide. These contacts exhibited the highest FC*_ij_*^app^ values, reaching several hundreds kcal/molÅ^2^, reflecting the strong covalent linkage between the sugar and the nucleobase that stabilizes the nucleoside. Two well-defined peaks were observed, corresponding to different C2–C1′ distances and interaction strengths: pyrimidines displayed shorter distances and higher FC*_ij_*^app^ values, whereas purines showed longer distances and consequently lower force constants (Fig. 3, upper left). Because these contacts are essentially independent of distance within the same nucleoside, they were represented by ENM springs with a constant force parameter *C*. The second class of interactions corresponds to “backbone” contacts involving P and C1′ beads, either within the same nucleotide or between subsequent nucleotides, thereby defining the CG sugar–phosphate backbone of the nucleic acid. The highest FC*_ij_*^app^ values (up to ∼150 kcal/molÅ^2^) were found for P–C1′ pairs in adjacent nucleotides, followed by weaker interactions for intra-nucleotide P–C1′ contacts, consecutive P–P pairs, etc. (Fig. 3, upper right). Unlike “intra-nucleoside” contacts, these interactions exhibited a clear distance dependence and were therefore parametrized using the distance-decaying law of equation (2). The third class, denoted “base-base” contacts, consists of C2–C2 interactions and captures specific nucleobase pairing and stacking. Two prominent peaks were identified in the FC*_ij_*^app^ distribution (Fig. 3, lower left). The first, at C2–C2 distances of 4.1–4.4 Å, corresponds to canonical CG and AT/AU base pairs. The second, at 6.7–7.0 Å, arises from GG contacts involved in stacking interactions within a β-hairpin structure present in the DNA MD dataset (Supplementary Fig. S4). FC*_ij_*^app^ values exceeded ∼50 kcal/molÅ^2^ for CG base pairs and GG stacking interactions, whereas AT/AU pairs displayed lower force constants, consistent with their smaller number of hydrogen bonds compared to CG pairs. These strong, localized interactions were represented by distance-independent springs characterized solely by the parameter *C*. Finally, all remaining bead pairs not assigned to the previous three classes were grouped in a fourth category of long-range, non-bonded contacts, here termed “*Van der Waals*” interactions (Fig. 3, lower right panel). In contrast to “intra-nucleoside” and “base–base” contacts, these interactions span a much broader range of distance-decaying force constants and were thus modeled using the distance-dependent spring law of equation (2).

**Figure 4.**
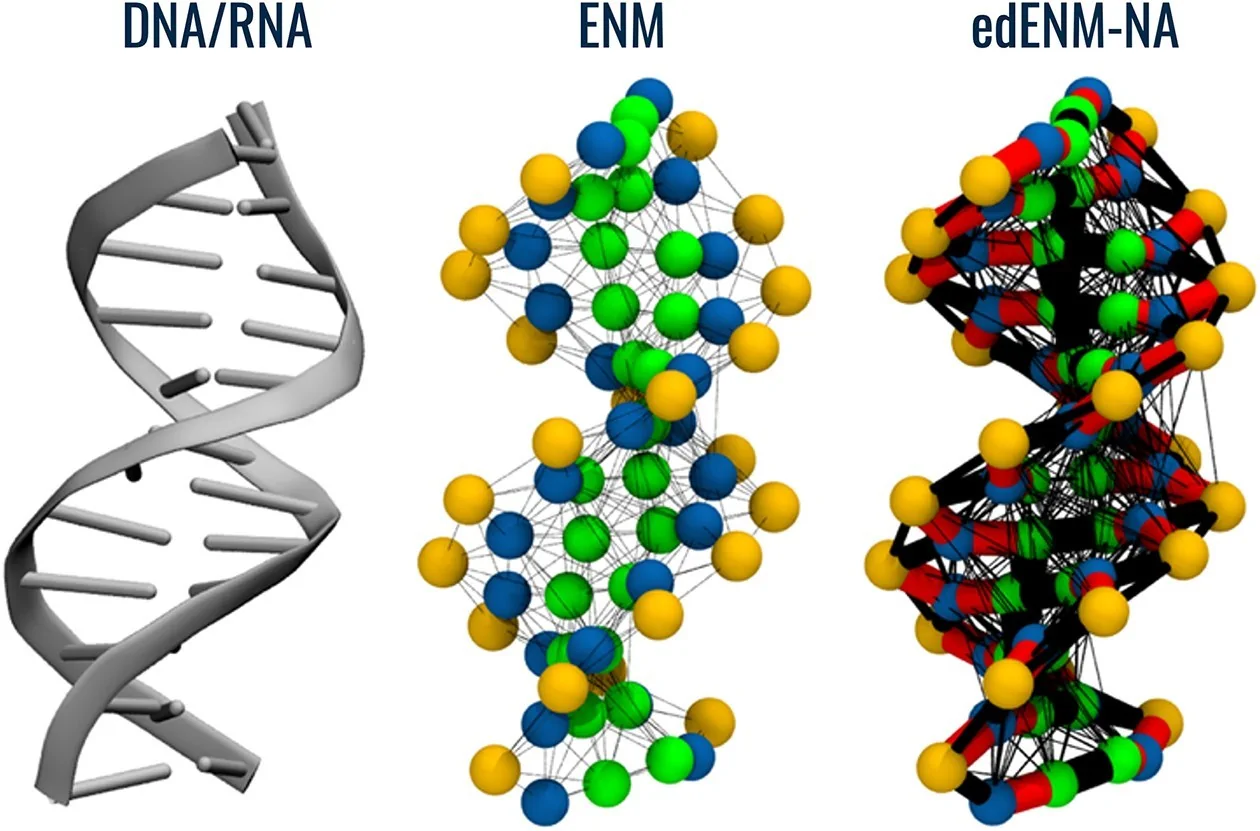
Elastic network topology for nucleic acids (in this case, a double-stranded DNA helix; PDB ID: 7XH3). The standard uniform-spring SBP-ENM from Pinamonti *et al.* [39] is compared to our refined edENM-NA. P, C1′, and C2 atoms are represented as spheres, with P atoms in yellow, C1′ atoms in blue, and C2 atoms in green. Springs are represented with black cylinders with diameters proportional to the spring constant values. In the edENM-NA, diameters have been saturated to a maximum value of 65 kcal/molÅ^2^, and spring constants exceeding this value (“intra-nucleoside” and “base–base” interactions) are represented as red cylinders.

The range of *C* and *n* parameter values that was employed in the random search refinement for the four interaction types is reported in Supplementary Table S5. An example of fitting interval based on minimum and maximum values for the “backbone” and “Van der Waals” distance-dependent interactions is shown in Supplementary Fig. S5. After refinement, the C2–C1′ “intra-nucleoside” interactions were associated with constant values of *C*_pyr_ = 290 kcal/(molÅ^2^) for pyrimidines, and *C*_pur_ = 120 kcal/(molÅ^2^) for purines. The C2–C2 “base-base” contacts for the strong CG and GG interactions were optimized with a constant value of *C*^bb^ = 65 kcal/(molÅ^2^). The interactions between P and C1′ atoms in the “backbone” were optimized with a distance-dependent law, with *C*^back^ = 20 and *n*^back^ = 2.8. Finally, all remaining “Van der Waals” contacts were modeled with *C*^VdW^ = 25 and *n*^VdW^ = 1. At the end of the edENM-NA parametrization, the optimal cutoff for “backbone” and “Van der Waals” distance-dependent interactions was set to 11 Å. Table 1 reports all contact types and the spring constants in the edENM-NA. A visual representation of the resulting edENM-NA topology is shown in Fig. 4 and compared to the traditional uniform-spring ENM [39].

**Table 1.** Contact classes and spring constants employed in edENM-NA, including those at the protein–nucleic acid interfaces within edENM-PNA

Model	Contact	Bead pair	Sub-class	Distance decay	*C*	*n*	Cutoff
edENM-NA	Intra-nucleoside	C2–C1′ (*S* = 0)	Pyrimidine	No	290	-	-
			Purine		120		
	Backbone	P–C1′+ P–P	-	Yes	20	2.8	11
	Base-base	C2–C2	CG (4.1–4.4Å)	No	65	-	-
			GG (6.7–7.0Å)				
	Van der Waals	P–C2 + C1′–C1′ + C2–C1′ (*S* > 0) + C2–C2 (not base-base)	-	Yes	25	1.0	11
edENM-PNA interface	Type-1	Polar/basic C_α_–P Acidic C_α_–C2	-	Yes	25	2.2	8
	Type-2	Not type-1	-	Yes	30	1.0	8

After finding the optimal spring network connectivity and spring parameters (Table 1), we compared the edENM-NA to the three-bead uniform-spring ENM proposed by Pinamonti *et al.* [39]. For sake of simplicity, the uniform-spring model from Pinamonti *et al.* [39] will be referred to simply as ENM in the following. First, we compared the performance of the ENM and edENM-NA in capturing the MD flexibility by comparing their NMs against the PCs of four different systems in the MD testing set (Supplementary Table S1). From this comparison, we observed that the proposed ED-based parametrization produces reasonable overlaps with MD simulation data, and it slightly increases the RMSIP agreement between the lowest-frequency NMs and the trajectory PCs in the testing set compared to the uniform-spring parametrization (Supplementary Fig. S6).

**Figure 5.**
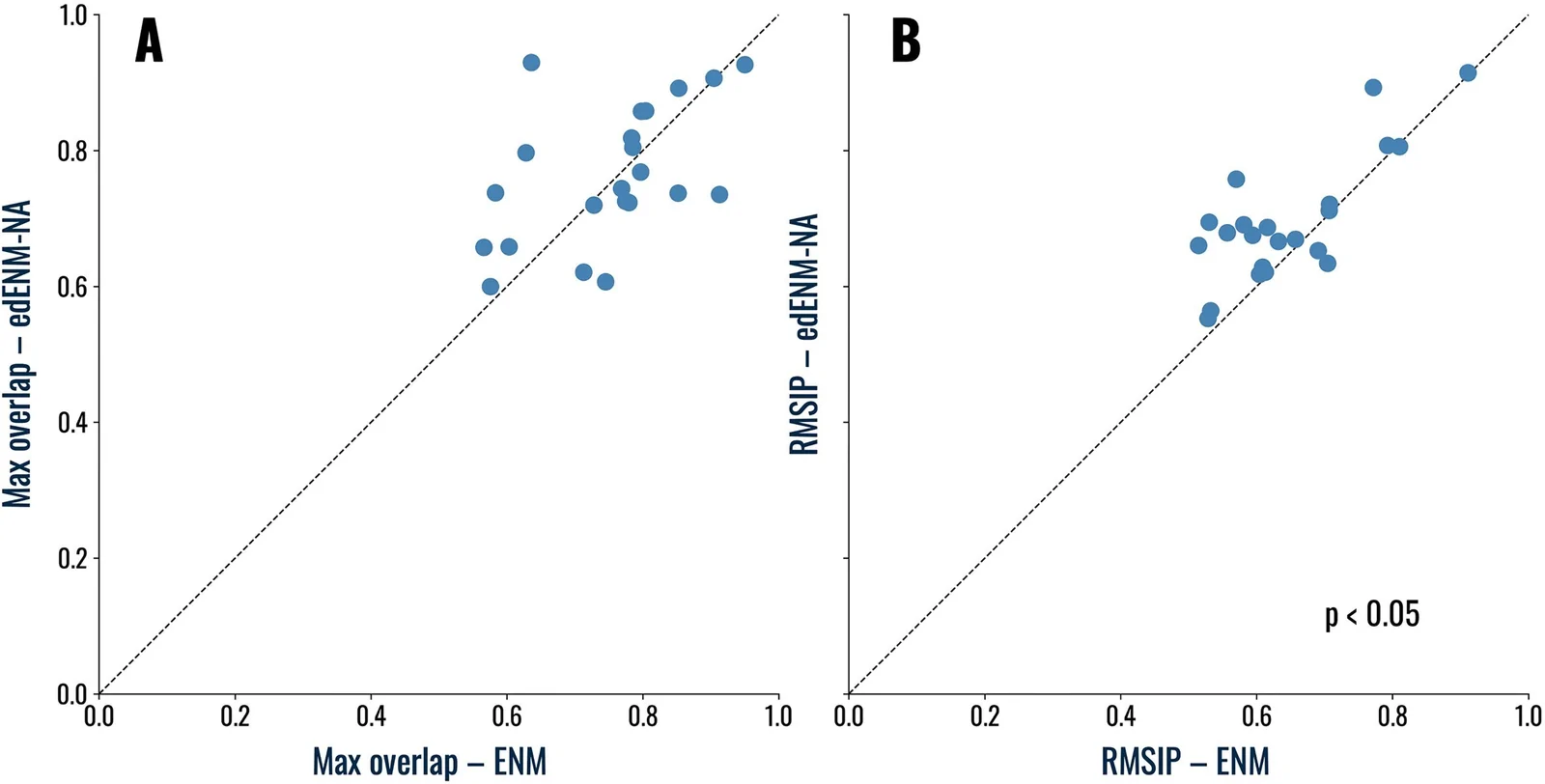
Comparison between edENM-NA and ENM in terms of ability to capture experimental PCs from the set of multi-model PDB structures of nucleic acids (Supplementary Table S2). (**A**) *O*_max_ for the set of first 3 NMs and experimental PCs. (**B**) RMSIP for the set of first 3 NMs and experimental PCs. *P*-values arising from a one-sided Wilcoxon test (edENM-NA > ENM) between *O*_max_ and RMSIP scores are 0.33 and 4 × 10^−4^, respectively (Supplementary Table S6).

Then, we compared the performance of the edENM-NA in terms of its ability to capture apparent motions in the testing dataset of DNA- and RNA-only multi-model PDBs (Supplementary Table S2) and compared it to the ENM. Both ENM and edENM-NA were generally able to describe the experimental PC motions via their low-frequency NMs (Supplementary Fig. S7 and Fig. 5). Average values of *O*_max_ and RMSIP scores across the multi-model testing dataset were >0.6 for both models (Supplementary Table S6), with maximum overlap scores reaching 0.9, suggesting very good agreements between experimental and predicted motions. Moreover, most of the *O*_max_ scores were found between NM1 to PC1 (Supplementary Table S6), indicating that the lowest-frequency NMs from simplified ENM topologies have the ability to capture the main directions of motion from the experimental ensembles.

**Figure 6.**
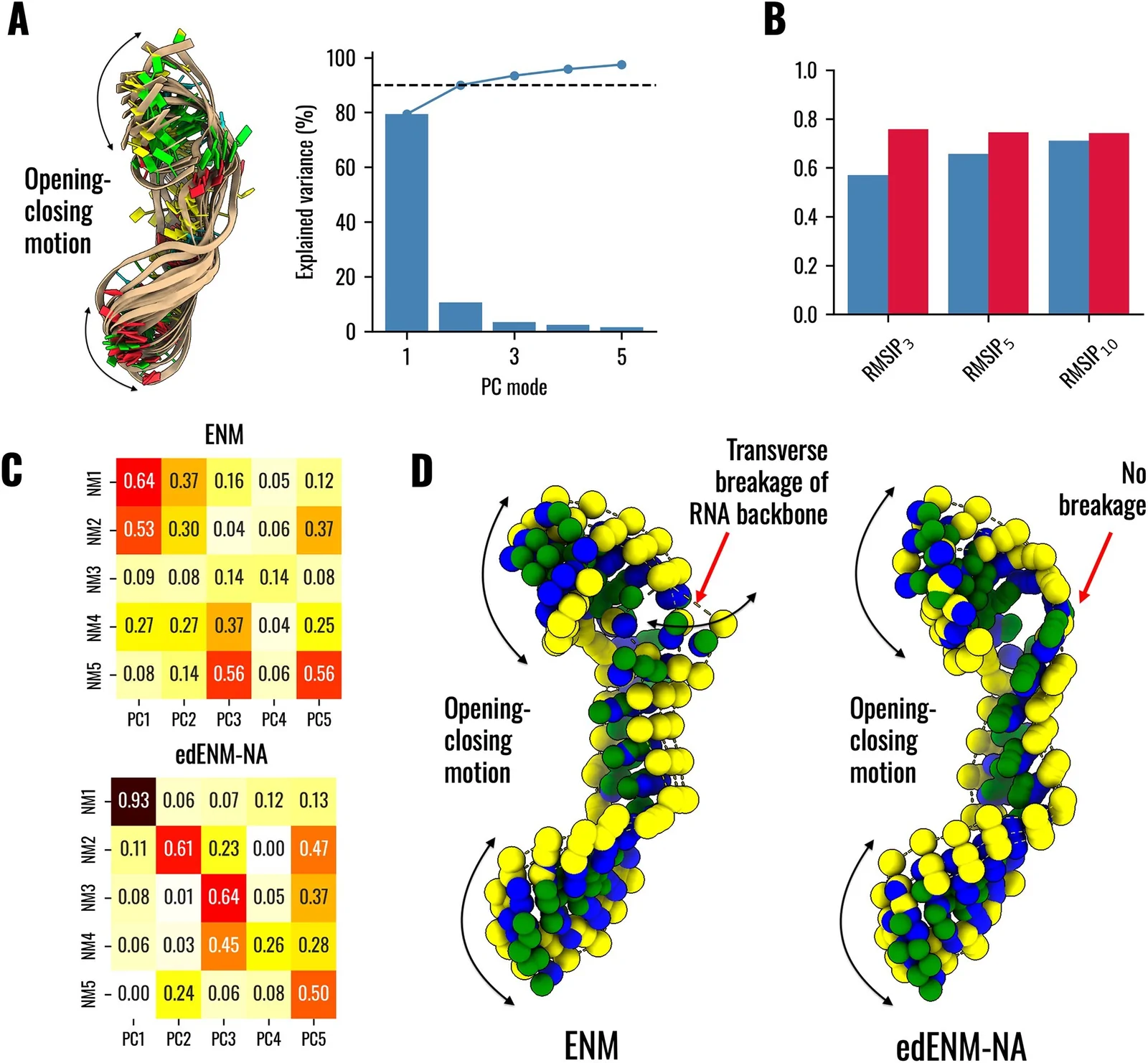
Comparison between ENM, edENM-NA, and PC modes from the NMR ensemble (PDB: 2PCV) of the U65 Box H/ACA small nucleolar RNA. (**A**) Ensemble of 20 models in the solution NMR ensemble used for PCA. The black arrows highlight the opening–closing motion apparent from the experimental ensemble, which dominates PC1. The graph shows the variance captured by each PC mode (blue bars), the cumulative variance of the first PC modes (continuous blue line), and a threshold of 90% variance (dashed black line). PC1 captures ∼80% variance and PC1 + PC2 together allow to capture ∼90% variance. (**B**) Comparison between ENM (blue bars) and edENM-NA (red) in terms of RMSIP scores (considering both 3, 5, and 10 modes) between NMs and experimental PCs. (**C**) Matrix of overlaps between the first 5 PC modes and the first 5 NMs from ENM and edENM-NA. Maximum overlaps are found between NM1 and PC1 and reach 0.64 for ENM and an astonishing 0.93 for edENM-NA. (**D**) Graphical representation of the motions associated with NM1 for ENM (left) and edENM-NA (right). Beads represent P (yellow), C1′ (blue), and C2 (green) positions across three deformed structures along the NM. Note how both models allow to capture the opening–closing motion observed in the NMR ensemble, but the uniform-spring ENM shows a “breakage” (pronounced transverse oscillation) of the RNA backbone, which is prevented in the edENM-NA due to stronger backbone interactions.

The performance of edENM-NA was satisfactory in terms of *O*_max_ scores, and in a few cases, the ED-based parametrization provided a much higher overlap compared to the ENM. For instance, the opening–closing motion in the NMR of the U65 Box H/ACA small nucleolar RNA (PDB: 2PCV) was captured with 0.64 similarity by NM1 of the ENM, and with a remarkable 0.93 by the edENM-NA (Fig. 6). We observed that the lower ENM similarity was due to an unrealistic transverse “breakage” of the RNA backbone in the dominant NM (Fig. 6D), which was not generated by edENM-NA due to its higher spring constants in the RNA backbone (see Fig. 4). Intriguingly, in a few cases, *O*_max_ scores were slightly higher when using the simpler ENM parametrization (Supplementary Table S6). For instance, the opening–closing motion related to the experimental PC1 of a III–IV–V three-way junction of VS ribozyme (PDB: 2MTJ) was captured with 0.74 similarity by NM1 of edENM-NA, while exhibiting a higher overlap (0.85) with NM2 of the simpler ENM (Supplementary Fig. S8). However, looking in more detail at this case, we observed that the lowest-frequency mode (NM1) of the uniform-spring ENM gave rise to an extremely localized oscillation involving a detachment of the C2 bead in G47 (Supplementary Fig. S8), making this dominant NM completely uncorrelated with experimental motions from the NMR ensemble. As a result, the RMSIP of the three lowest-frequency modes was still higher in the optimized edENM-NA (0.67) than in the ENM (0.59), as evident for most cases from Fig. 5B.

When the performance of the two models was evaluated across the entire dataset of multi-model structures, we found that *O*_max_ values in edENM-NA were on average greater than in the ENM (Supplementary Table S6), even though the slightly positive difference (∼ +0.015) was not found to be statistically significant (*P*-value ∼ .33). On the other hand, a clear significant difference was observed when comparing the lowest-frequency NM sub-spaces through RMSIP scores, with edENM-NA significantly better performing (∼ +0.05, *P*-value < .0005) than the uniform-spring parametrization (Supplementary Table S6). Taken together, these results suggest that, while correlations between individual NMs and experimental motions might not necessarily be higher in all cases, our ED-based model tends to generate more realistic harmonic motions, with no evident backbone breakage or extremely localized oscillations, and exhibits significantly higher values of sub-space similarity (RMSIP) with experimental PCs.

### Parametrization and testing of edENM-PNA

The distribution of protein–nucleic acid FC*_ij_*^app^ values as a function of the inter-bead distances was computed using the corresponding MD dataset for protein–nucleic acid systems in Supplementary Table S1. The relationship between distances *d_ij_^a^* and FC*_ij_*^app^ was inspected considering all possible combinations of contacts between different protein residues and nucleic acid bead types. Protein amino acids were divided according to their chemical nature, i.e. acidic, basic, polar, and hydrophobic. From the FC*_ij_*^app^–*d_ij_^a^* plots, we observed that the interactions between P atoms with polar or basic residues in proteins showed a marked distance-dependent decay with *n* > 1, while the remaining pairs had *n* closer to 1 (Supplementary Fig. S9). In case of C1′ beads, no interaction couple showed a clear decay with *n* > 1 (Supplementary Fig. S10), while for C2 beads, we observed exponents >1 in the case of acidic residues (Supplementary Fig. S11). Thus, we defined two types of interaction to describe the network of protein–nucleic acid contacts, i.e. “type-1” and “type-2” interactions, both following a distance-dependent decay law as for equation (2). The former were defined for interactions between P beads with polar and basic residues, as well as interactions between C2 beads with acidic residues. The latter were used to describe all remaining connections between C_α_ atoms of the protein and nucleic acid beads. The parameters for “type-1” and “type-2” protein–nucleic acid interactions were fitted based on the MD-derived FC*_ij_*^app^–*d_ij_^a^* data. The range of parameters explored in the random search and in the subsequent refinement is reported in Supplementary Table S7. After refinement and optimization using NMR models of protein–nucleic acid complexes (Supplementary Table S3), we found optimal values *n*^type-1^ = 2.2, *C*^type-1^ = 25, and *C*^type-2^ = 30 (*n*^type-2^ = 1), with an optimal protein–nucleic acid interaction cutoff of 8 Å (Table 1). Regarding the topology of the protein–nucleic acid interface, differently from the standard ENM approaches, we introduced a limit in the number of springs connecting protein residues and nucleotides. For each pair of protein–nucleic acid residues, we considered only one elastic connection, specifically between the C_α_ atom of the protein and the closest bead in the nucleotide within the interaction cutoff (Supplementary Fig. S12A and B). This criterion was implemented to avoid excessive stiffening at the protein–nucleic acid interface.

We compared the NMA results arising from edENM-PNA to those obtained with a standard uniform-spring ENM, where all springs have equal force constants. To identify the contribution of the new nucleic acid parametrization, we also considered a “hybrid” model, that we call here edProt, which is a combination of the edENM force-field [29] for the protein and the uniform-spring ENM parametrization of Pinamonti *et al.* [39] for the nucleic acid (see Supplementary Fig. S12C). The performance of these three models (edENM-PNA, ENM, and edProt) was assessed in terms of their capability to capture ED modes from a test set of three MD simulations (Supplementary Table S1), experimental PCs from a set of NMR complexes (Supplementary Table S3), as well as experimental PCs from conformational ensembles (Supplementary Table S4). For two [81, 82] out of the three testing MD simulations, both edProt and edENM-PNA provided greater RMSIP values than ENM, with edENM-PNA performing generally slightly better than edProt (Supplementary Fig. S13), confirming the advantage of using an MD-refined parametrization for both the protein and the nucleic acid to better capture motions of the system. Surprisingly, in one of the three simulations [83], the edENM-PNA/edProt performance was lower. In this case, the trajectories focused on the movements of disordered histone tails in a nucleosome complex, which are hardly modeled by any ENMs, as confirmed by low values of RMSIP in all three models (<0.4).

**Figure 7.**
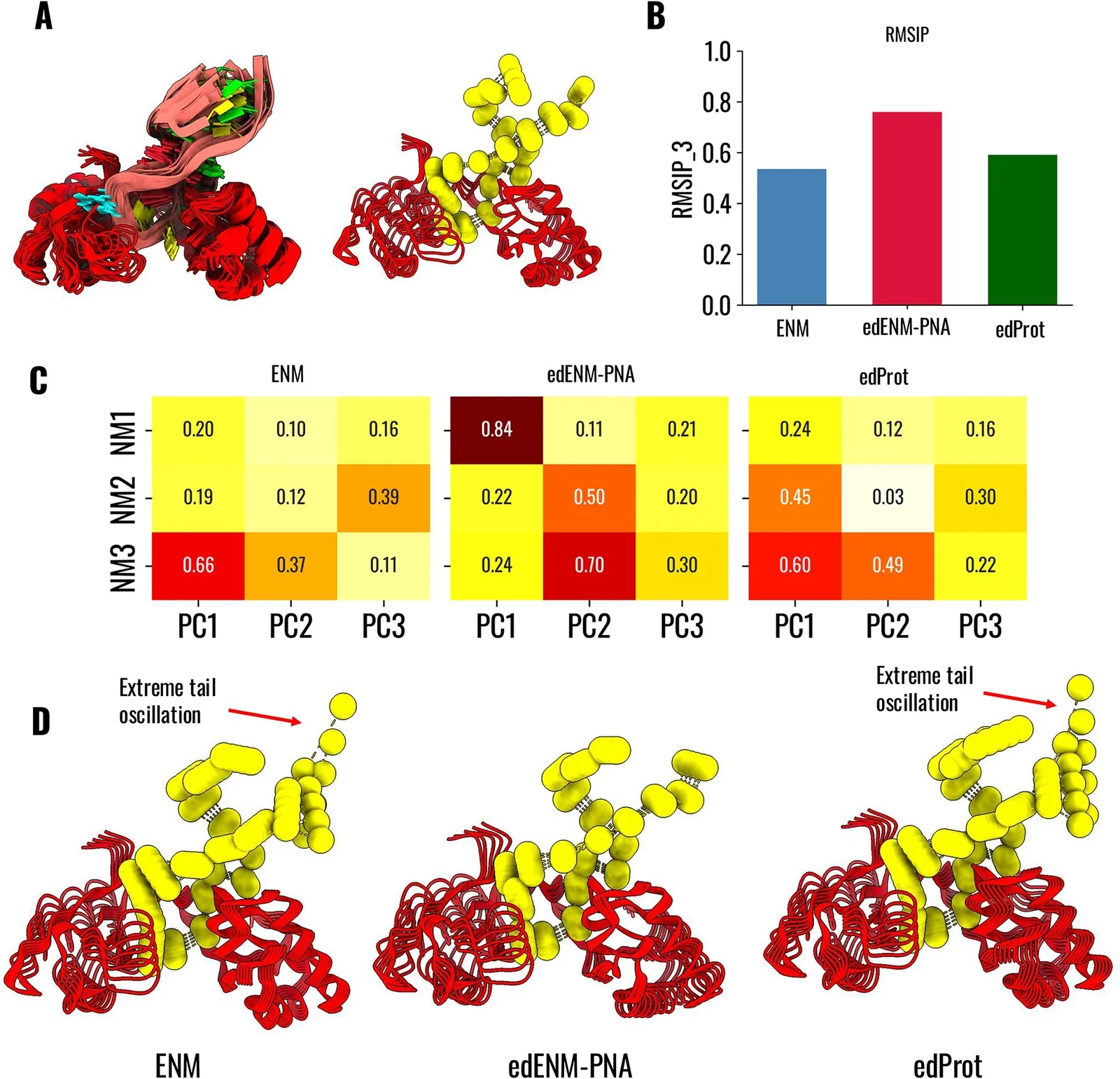
Comparison between ENM, edENM-PNA, and edProt and PC modes from the NMR ensemble (PDB: 1FJE) of the nucleolin RBD12 in complex with RNA. (**A**) Ensemble of 19 models (left) in the solution NMR used for PCA and direction of PC1 eigenvector (right), projected on the protein (red cartoon) and the RNA P-atoms (yellow spheres). (**B**) Comparison between ENM, edENM-PNA, and edProt in terms of RMSIP scores between the first three NMs and the first three experimental PCs. (**C**) Matrix of overlaps between the first three PC modes and the first 3 NMs from ENM, edENM-PNA, and edProt. (**D**) Graphical representation of the motions associated with NMs with the highest overlap with PC1, i.e. NM3 for ENM (left), NM1 for edENM-PNA (center), and NM3 for edProt. Note how all the models properly describe the conformational flexibility in the protein, but ENM and edProt introduce extreme oscillations in the RNA tail, which is not observed using the ED-refined nucleic-acid parametrization.

For the set of 21 NMR protein–nucleic acid complexes (Supplementary Table S3), edENM-PNA was found to provide higher similarity scores to the experimental PCs (average *O*_max_: 0.59, average RMSIP: 0.51) compared to the uniform-spring ENM (*O*_max_: 0.52, RMSIP: 0.45) and edProt (*O*_max_: 0.45, RMSIP: 0.49, Supplementary Table S8). The comparison between edENM-PNA and ENM showed significantly improved scores in edENM for both *O*_max_ and RMSIP (*P*-value < .05; Supplementary Table S9 and Supplementary Fig. S14), confirming the higher performance of the ED-based parametrization for both the protein and the nucleic acid. On the other hand, statistically significance differences concerning edProt were more difficult to interpret. edENM-PNA was not performing significantly better than edProt for RMSIP (*P*-value: ∼ .34), while it was close to a significant improvement for *O*_max_ (*P*-value: ∼ .08). On the other hand, edProt had a clear significant improvement over ENM in terms of RMSIP (*P*-value: ∼ .01) but not for *O*_max_ (*P*-value: ∼ .09). ENM was never found to perform better than either edProt or edENM-PNA (*P*-values > .9). Taken together, these results suggest that an ED-based parametrization both at the protein and nucleic acid level significantly improves the performance over the uniform-spring ENM, while the significancy at the level of protein and nucleic acid components separately might depend on a case-by-case basis. For example, edENM-PNA clearly outperformed both ENM and edProt in describing the PCs from the NMR ensemble of nucleolin RNA-binding domain 12 (RBD12) in complex with RNA (PDB: 1FJE). The experimental motion along PC1 was captured by edENM-PNA with 0.84 similarity with NM1, while the maximum agreement with ENM and edProt was found with NM3 with 0.66 and 0.60 similarity, respectively (Fig. 7). Looking at the predicted oscillations, we observed that the stronger nucleic acid backbone of edENM-PNA allows to avoid the generation of extreme displacements in the RNA tail that is observed in ENM and edProt (Fig. 7D). Together with the previous examples shown in Fig. 6 and Supplementary Fig. S8, this confirms that the ED-based parametrization improves the stability of the NMs in nucleic acids, avoiding backbone ruptures and extremely localized fluctuations.

We also assessed the similarity of the NMs from edENM-PNA, ENM, and edProt to the experimental PC eigenvectors arising from a curated and highly heterogeneous set of protein–nucleic acid ensembles from X-ray and cryo-EM conformations (Supplementary Table S4). In this case, we computed overlaps and RMSIPs considering the NMs from each individual PDB in the ensemble, and Supplementary Table S10 reports average and maximum *O*_max_ and RMSIP scores for each ensemble. Overall, the similarity scores in edENM-PNA were found to surpass (∼ +0.01–0.04) those from the ENM and edProt, confirming the slightly better performance of edENM-PNA in capturing the main directions of motions extracted from experimental ensembles. When assessing the statistical significance of such differences, these were found to be highly significant at the level of individual PDBs (*P*-values < 1 × 10^−4^, Supplementary Table S11) for both *O*_max_ and RMSIP (Supplementary Fig. S15). Due to the highly uneven number of PDB structures across different ensembles (Supplementary Table S4), we also assessed the statistical significance of these differences when considering aggregated *O*_max_ and RMSIP scores for each ensemble, aggregating by average and maximum values. In this case, the statistical significance of higher edENM-PNA scores was found to decrease both for average (Supplementary Table S12 and Supplementary Fig. S16) and maximum scores (Supplementary Table S13 and Supplementary Fig. S17). edENM-PNA was still found to perform significantly better than edProt (*P*-values < .05), suggesting the higher performance of employing an ED-based parametrization for the nucleic acid when coupled with an ED-based parametrization of the protein. ENM was never found to be significantly better than edENM-PNA (*P*-values ∼ .8) or edProt (*P*-values ∼0.4–0.9), while the opposite hypotheses (edENM-PNA > ENM and edProt > ENM) were associated with *P*-values of ∼ .2 for edENM-PNA and ∼0.05–0.6 for edProt.

**Figure 8.**
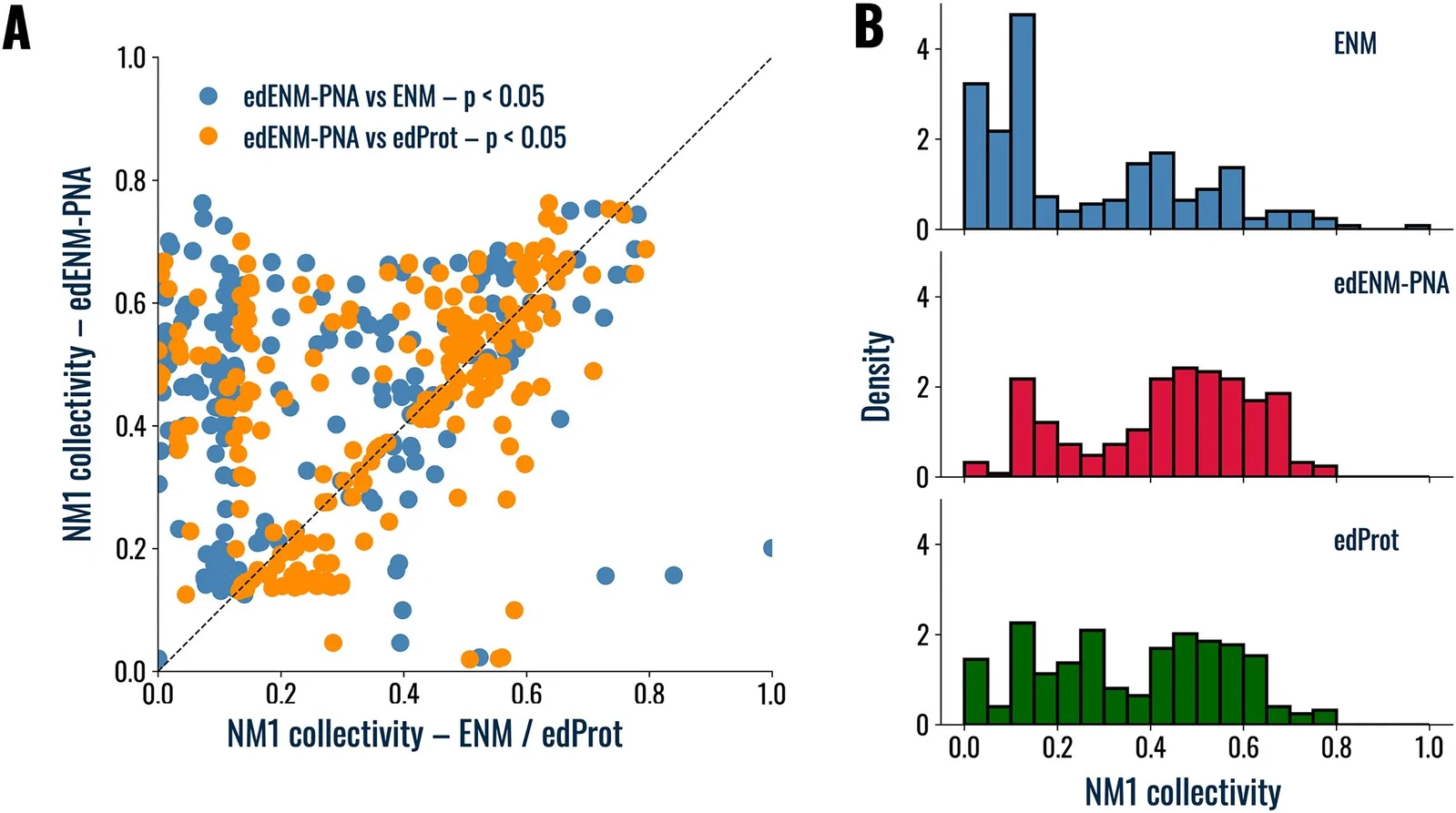
Motion collectivity for the lowest-frequency normal mode (NM1) evaluated from edENM-PNA, ENM, and edProt. (**A**) Scatterplot showing the comparison between NM1 collectivity between edENM-PNA and ENM (blue points) or edENM-PNA and edProt (orange points). (**B**) Distribution of NM1 collectivity values for ENM (blue), edENM-PNA (red), and edProt (green). *κ* values for NM1 averaged across the entire dataset are 0.436 ± 0.185 for edENM-PNA, 0.363 ± 0.201 for edProt, and 0.262 ± 0.222 for ENM. *P*-value analysis arising from one-sided Wilcoxon tests are reported in Supplementary Table S14.

For the entire dataset of PDBs of protein–nucleic acid ensembles, we also computed collectivity scores *κ* of the lowest-frequency normal mode (NM1) predicted by edENM-PNA, edProt, and ENM. NM1 represents the softest, lowest-energy mode and is generally expected to be highly collective. This analysis revealed that edENM-PNA consistently provides higher *κ* values (∼0.44 on average) than both edProt (∼0.36) and ENM (∼0.26), with highly significant differences in both cases (*P*-values < 1 × 10^−4^; Supplementary Table S14). As shown in Fig. 8, ENM can produce extremely localized vibrations, e.g. *κ* < 0.1, in the most dominant NM, while the ED-based parametrization generates consistently more collective motions, *κ* > 0.5. Taken together, these results confirm that edENM-PNA enhances the collectivity of the lowest-frequency harmonic vibrations.

### Exploring conformational transitions with eBDIMS

After assessing the performance of the edENM force-field for nucleic acids and protein–nucleic acid complexes via NMA, the edENM-PNA parameters were integrated into the eBDIMS2 algorithm [50], to be able to simulate transition pathways not only for proteins, but also for RNA, DNA, and any macromolecular complex (eBDIMS2-PNA).

**Figure 9.**
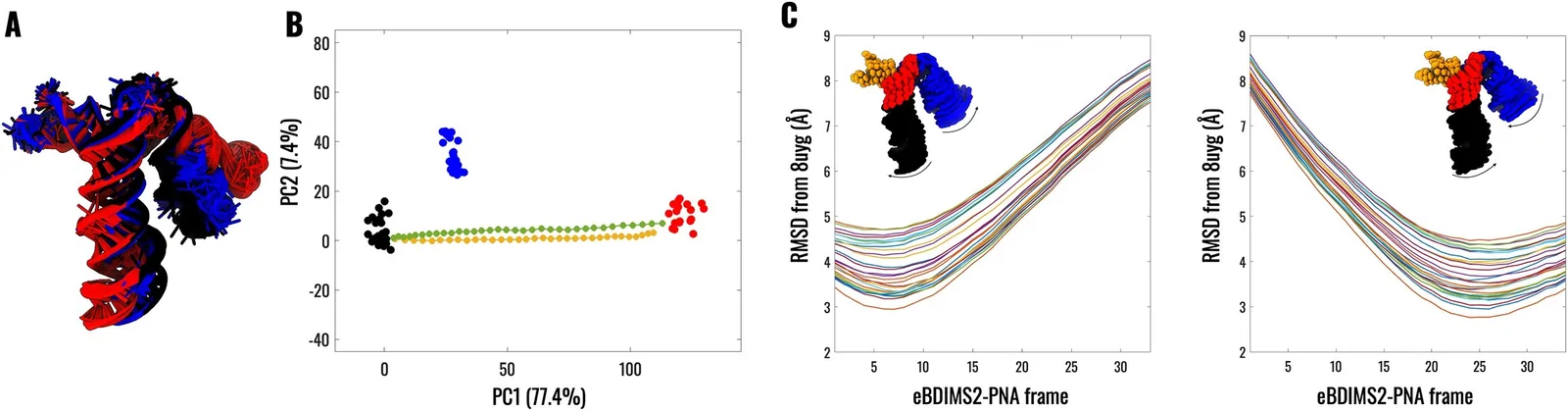
Conformational heterogeneity and eBDIMS2-PNA transition pathways of the SL5 RNA from *BtCoV-HKU5* (∼40 kDa). (**A**) Cryo-EM SL5 conformations clustered into closed (black; PDB: 8UYE), open (red; 8UYJ), and intermediate (blue; 8UYG) states [84]. All deposited models within each PDB entry are shown to illustrate positional variability. (**B**) PCA projection of the experimental conformational ensemble and eBDIMS2-PNA transition pathways (closed-to-open in orange; open-to-closed in green). (**C**) RMSD between eBDIMS2-PNA frames and the individual models of the intermediate conformer (8UYG). Each line refers to an individual model in the PDB file. Insets illustrate the hinge-bending motion of the SL5a arm along the eBDIMS2-PNA trajectory, with P, C1′, and C2 atoms represented as spheres, and SL5 segments colored as follows: SL5-stem (black), SL5a (blue), SL5b (orange), SL5c (red).

A recent cryo-EM study resolved the tertiary folds of the SL5 genomic RNA elements in six coronaviruses, revealing a conserved T-shaped architecture composed of two perpendicular coaxial stacks formed by the stem–SL5c and SL5a–SL5b domains [84]. In two β-coronaviruses, *BtCoV-HKU5* and *MERS*, this architecture exhibits a pronounced conformational heterogeneity, characterized by a hinge-like motion originating from the SL5a internal loop [84]. Structural models of *BtCoV-HKU5* cluster into closed, open, and intermediate conformations (Fig. 9A). PCA of the experimental ensemble shows that the dominant motion—a large-scale hinge-bending transition between closed and open states—is captured by the first PC, accounting for ∼77% of the variance (Fig. 9B). NMA yielded very high overlaps between the lowest-frequency edENM-NA modes and PC1 (*O*_max_ > 0.8), with edENM-NA slightly outperforming the uniform-spring parametrization (Supplementary Table S6). To model the full hinge-bending transition (RMSD ∼10.5 Å) beyond the directionality of a small-scale harmonic vibration, we used eBDIMS2-PNA to simulate both the closed-to-open and open-to-closed pathways. All simulations converged to the target conformations with final RMSDs below 1Å (Fig. 9B) and consistently tended to approach the cluster of experimentally observed intermediate conformers before converging to the targets (Fig. 9C), demonstrating that eBDIMS2-PNA can now generate conformational transitions not only in proteins [49, 50], but also in nucleic acids.

**Figure 10.**
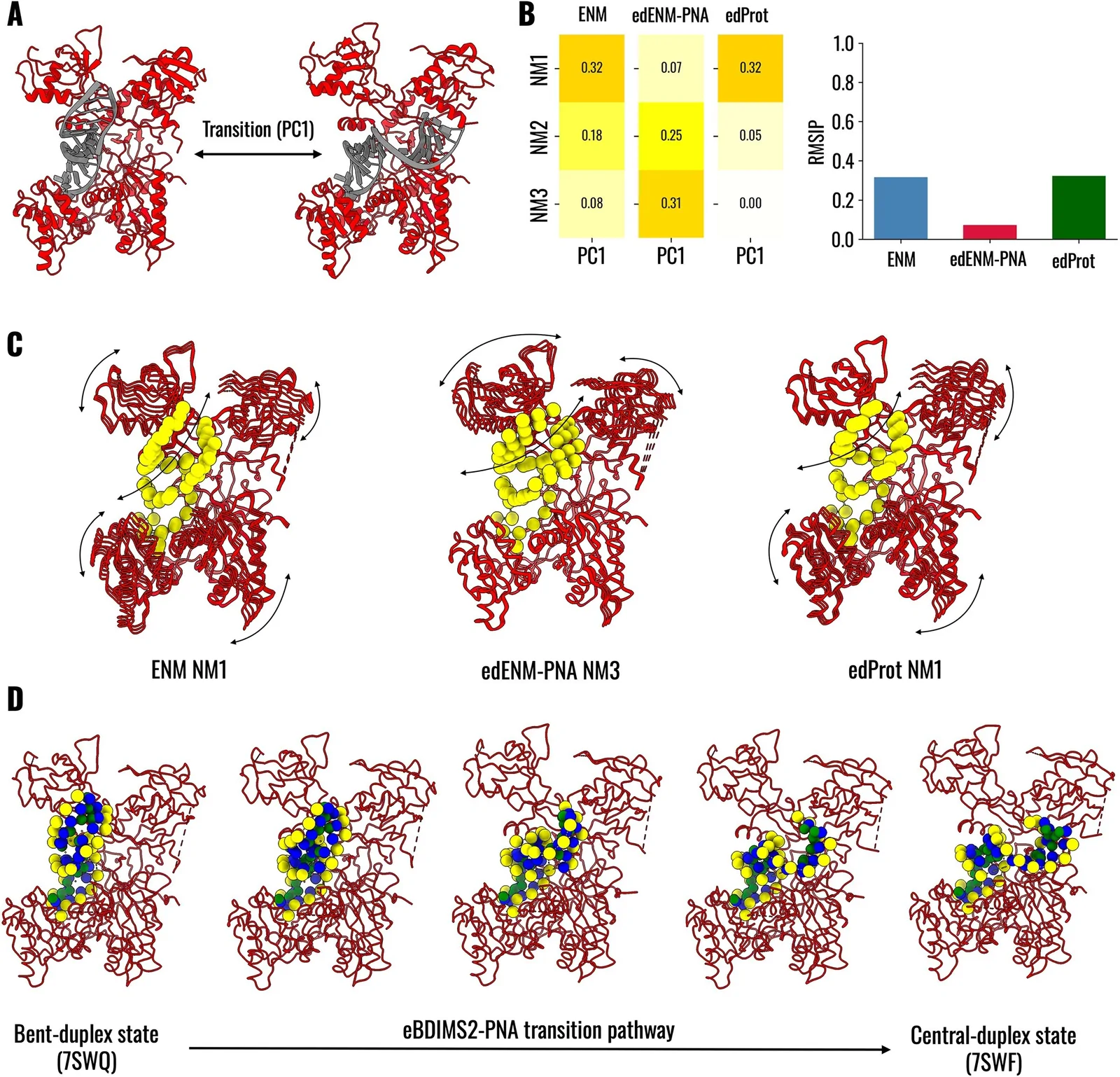
Conformational change and eBDIMS2-PNA transition pathways of the *A. Thaliana* Argonaute10 (AtAgo10)–guide–target RNA complex (∼120 kDa). (**A**) Cryo-EM bent-duplex (PDB: 7SWQ, left) and central-duplex (7SWF, right) conformations of the AtAgo10–guide–target complex [11], highlighting the motion of RNA chains within the complex (PC1). (**B**) Comparison between ENM, edENM-PNA, and edProt in terms of overlap and RMSIP scores between the first three NMs and conformational transition (PC1). (**C**) Graphical representation of the motions associated with NMs with the highest overlap with PC1, i.e. NM1 for ENM (left), NM3 for edENM-PNA (center), and NM1 for edProt (right). (**D**) Snapshots of AtAgo10–guide–target complex intermediates along the transition pathways simulated with eBDIMS2-PNA from the bent-duplex to the central-duplex state, highlighting intermediate RNA positions.

Another recent cryo-EM study identified two functional states of *A. thaliana* Argonaute10 (AtAgo10) in complex with guide and target RNAs (Fig. 10): a “slicing”-incompetent bent-duplex state and a “slicing”-competent central-duplex state [11]. The transition between these states involves coordinated rearrangements of the PAZ and N domains [11] and a localized reorganization of the bound RNA duplex. PCA of the two-state ensemble, including both protein and RNA components, yielded a single meaningful PC describing this transition, which spans a relevant structural change (RMSD ∼5.5 Å) but is weakly collective (*κ* ∼ 0.07), as it mainly involves the repositioning of the RNA strands within the protein scaffold. Consistent with its localized and complex nature, this transition was poorly captured by NMA, with low overlaps between NMs and the transition vector (*O*_max_ ∼ 0.3), as low-frequency modes primarily describe collective motions of the whole protein–RNA complex (Fig. 10B and C). In contrast, eBDIMS2-PNA could successfully be used to provide a mechanistic view of the complex motion describing the repositioning of the two RNA strands from the bent- to the central-duplex state (Fig. 10D), reaching full convergence to the target conformation with a final RMSD of ∼0.4 Å. These results indicate that, although harmonic vibrational modes may fail to describe highly localized conformational changes [28, 33], ENM-based algorithms like eBDIMS2-PNA can still allow to capture the underlying (anharmonic) transition pathways, provide mechanistic insights, and generate physically plausible conformational intermediates.

**Figure 11.**
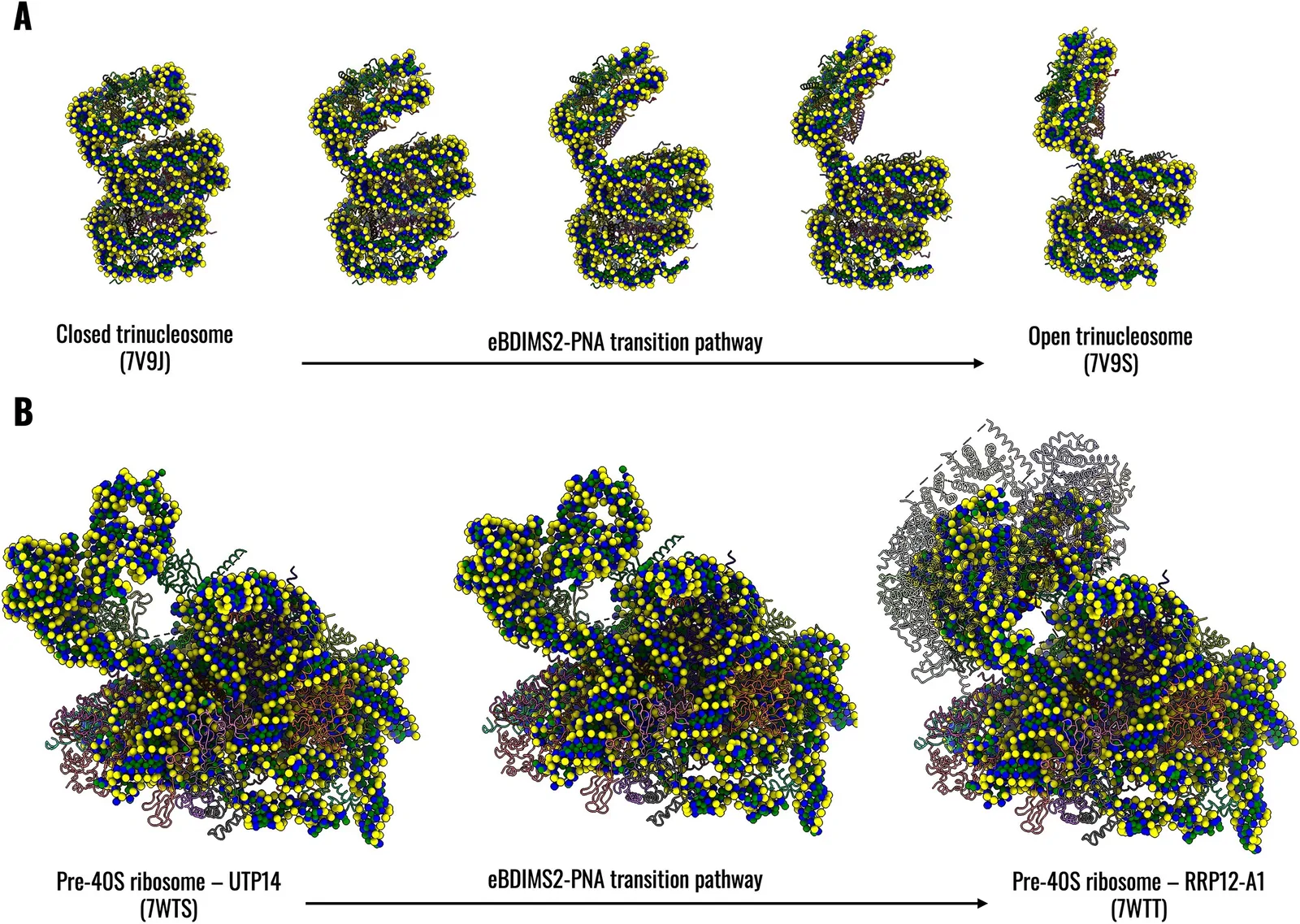
eBDIMS2-PNA transition pathways of the (**A**) *H. sapiens* telomeric tri-nucleosome (∼ 560 kDa) from closed columnar conformation (PDB: 7V9J) to open conformation (7V9S), highlighting the large-scale unstacking of one nucleosome; (**B**) *H. sapiens* pre-40S ribosomal subunit (∼ 1.3 MDa) from UTP14 (7WTS) to RRP12-A1 state (7WTT), highlighting the hinge-bending motion of the 3′ domain of the large 18S rRNA and its stabilization into the larger assembly upon binding with the additional ribosomal proteins.

As we recently demonstrated for protein systems, eBDIMS2 can be efficiently scaled to explore conformational transitions in macromolecular assemblies reaching the MDa scale [50]. Here, we further extend the applicability of the method to heterogeneous complexes composed of multiple proteins and long nucleic acid chains. The first example concerns a higher-order telomeric chromatin, which has been recently shown by cryo-EM to adopt columnar architectures characterized by tight nucleosome stacking [9]. Notably, these multi-nucleosome assemblies also populate alternative, more open conformational states, in which a single nucleosome can disengage from the stack and flips outward [9]. Figure 11A illustrates structural intermediates generated by eBDIMS2-PNA along the large-scale (RMSD ∼ 25 Å) closed-to-open transition of a telomeric tri-nucleosome. The resulting pathway revealed a highly cooperative rearrangement involving rigid-body translations and rotations of the moving histone core, coupled with DNA hinge-bending and twisting motions, highlighting the complex mechanical coupling required to achieve the column unstacking. The second example addresses the nucleoplasmic maturation of a pre-40S ribosomal subunit, for which recent cryo-EM studies have identified multiple assembly intermediates [10]. In an early nucloplasmic state (UTP14), the 3′ major domain of the 18S rRNA remains highly flexible and loosely connected to the remaining of the ribosomal body. In the subsequent RRP12-A1 state, the recruitment of additional assembly factors stabilizes the particle and repositions the 18S rRNA into a more stable complex [10]. eBDIMS2-PNA simulations bridging these two end states revealed a pronounced hinge-bending motion of the 18S rRNA (Fig. 11B), in which most of the ribosomal subunit behaves quasi-rigidly while the rRNA pivots about a localized hinge region within the nucleic acid strand. This motion provides a mechanism for head–body reorganization during nuclear maturation, illustrating how localized RNA flexibility can trigger larger conformational changes in assemblies such as ribosomal particles.

## Discussion

Here, we have shown that an MD-parametrized elastic network framework can capture the collective dynamics of DNA, RNA, and heterogeneous protein–nucleic acid complexes, extending the edENM approach previously validated for proteins to the full landscape of nucleic acid-containing biology. Prior efforts to develop ENMs for nucleic acids were largely focused on RNA systems [36, 38, 39], with comparatively limited attention devoted to DNA or unified descriptions. Early efforts explored the impact of CG strategies—such as the number and spatial placement of beads per nucleotide—on ENM performance, primarily evaluating their ability to reproduce atomic fluctuations or predict conformational changes [36]. More recently, ENMs for protein–nucleic acid complexes have been proposed in which parameter optimization was guided by maximizing the correlation between experimental and computed B-factors [40]. However, the use of B-factors as an optimization target remains controversial, as these are strongly influenced by crystal packing effects [43, 44, 85]. In contrast to the extensive methodological refinement achieved for protein ENMs [34], the development of optimized and transferable ENMs for nucleic acids has remained comparatively underexplored.

Rather than targeting local fluctuations and thermal motion, our parametrization protocol is explicitly designed to enhance the model’s ability to capture collective conformational changes observed in MD and experimental ensembles. In doing so, we have extended our previously developed edENM framework for proteins [29] to nucleic acids and further to heterogeneous protein–nucleic acid assemblies. By benchmarking the method across a diverse set of experimental structures obtained by NMR, X-ray crystallography, and cryo-EM, and by direct comparison with a simpler (uniform-spring) ENM parametrization, we found that the proposed model achieves on average improved agreements with the experimental motions (Fig. 5). Moreover, it yields more collective NMs (Fig. 8) and suppresses unphysical local backbone ruptures (Figs 6 and 7) that arise from overly weak or poorly connected spring networks. Moreover, the integration of the optimized edENM-PNA into the eBDIMS2 framework [49, 50] enabled us not only to assess harmonic motions, but also to explore large-scale anharmonic transitions in a wide range of nucleic acid–containing systems, from isolated RNA folds to MDa-scale assemblies. eBDIMS2-PNA could generate smooth transition pathways connecting experimentally resolved end states, providing structural intermediates and mechanistic insights even when the underlying motions were highly complex, such as the complex RNA rearrangement in AtAgo10 that could be poorly described by harmonic motions (Fig. 10). Thanks to the quasi-linear scaling of eBDIMS250, the approach could also be applied to larger assemblies, as illustrated by the simulations of the telomeric tri-nucleosome and pre-40S ribosomal subunit (Fig. 11). Together, these results demonstrate that the proposed edENM-PNA can naturally extend the eBDIMS2 framework to nucleic acid and protein–nucleic acid systems, enabling the investigation of biologically relevant conformational transitions across a broad range of sizes and molecular architectures.

A major strength of the proposed framework lies in the intrinsic efficiency and interpretability of ENMs. ENMs provide a highly simplified yet physically grounded representation of macromolecular dynamics [86], allowing conformational variability to be explored at a fraction of the cost of MD simulations, while still capturing dominant directions of motions [22, 80]. Across the systems examined here, low-frequency NMs exhibited high overlaps with experimentally captured conformational changes, in many cases even higher than 0.90 (Fig. 6), underscoring the ability of topology-driven models to capture biologically relevant motions, without requiring explicit chemical detail. It is well known that the performance of ENMs is strongly dictated by the network topology [31, 32, 34, 87, 88], rather than the fine-tuning of force constants. This inherently limits the extent to which parameter optimization can yield extreme gains in performance. Within this context, the improvements introduced by the present parametrization are nonetheless meaningful, as they address the main shortcomings of simpler models, including the suppression of unphysical backbone ruptures and overly localized modes (Figs 6–8). By promoting more collective motions and enforcing a stronger elastic coupling along the nucleic acid backbone, edENM-NA and edENM-PNA provide a more realistic description of collective conformational changes. Notably, the most pronounced performance gains were observed for nucleic acid systems, particularly from NMR ensembles (Fig. 5), which are expected to reflect intrinsic dynamical fluctuations. This is consistent with the vibrational nature of ENMs and contrasts with protein–nucleic acid assemblies, where conformational differences from structural ensembles may not be only dictated by vibrational motions, but also by more complex, localized transitions—such as the RNA rearrangement observed in AtAgo10 (Fig. 10). Taken together, these observations highlight both the strengths and domains of applicability of ENM-based approaches.

Despite the performance of the proposed modeling framework, several general limitations should be acknowledged. First, the development and validation of the model were constrained by the limited availability of structural datasets displaying large-scale conformational variability in nucleic acids and protein–nucleic acid systems. In total, our benchmarking relied on ~60 different macromolecular systems spanning both DNA, RNA, and protein–nucleic acid assemblies. While a higher number of available test cases would have strengthened the statistical significance of our validation, this limitation reflects a broader imbalance in structural biology, in which nucleic acids remain substantially underrepresented compared to proteins in public repositories such as the PDB [51]: currently, only ∼2% of PDB entries are nucleic acid-only, ∼6% comprise protein–nucleic acid systems, while ∼86% contain only protein entities. This scarcity is further highlighted by the difficulty of constructing coherent structural ensembles for complexes containing nucleic acids: even when complexes are captured in multiple conformational states, the associated nucleic acid components often differ in sequence or length, as in the case of RNaseIII–RNA complexes [49, 89], limiting their use in ensemble-based analyses. This limitation is not specific to the present framework but rather affects all computational approaches aimed at modeling nucleic acid dynamics. As cryo-EM and related structural biology methods continue to expand the structural coverage of nucleic acid-containing systems [90–92], increasingly rich and diverse conformational datasets are expected to become available, which will enable more extensive benchmarking, validation, and application of frameworks such as the one proposed here.

A second important limitation concerns the structural resolution of CG models. While vibrational modes and eBDIMS2-generated trajectories can provide valuable mechanistic insights into conformational changes at the CG (three-bead) level, approaches for reconstructing any CG model at atomistic resolution are still lacking for nucleic acids. Several reliable algorithms exist for CG-to-all-atom reconstruction of proteins, including cg2all [93], PULCHRA [94], etc., but tools for nucleic acids and protein–nucleic acid assemblies remain underdeveloped. Addressing this gap will be essential to reintroduce proper atomistic details to CG models containing nucleic acids for further downstream applications, such as MD simulations of conformational intermediates, interactions with small molecules, etc. This limitation, again, reflects a broader methodological imbalance in the field, where computational advances in structure prediction and conformational sampling have been overwhelmingly driven by protein-focused approaches, including deep-learning methods inspired by AlphaFold [95–99]. Some progress has been recently made for RNA [100–102]; however, the level of maturity and integration seen for protein systems has not yet been achieved for nucleic acids. The development of robust CG-to-atomistic reconstruction tools for nucleic acids represents the single most important methodological gap limiting a large-scale utility of edENM-PNA and eBDIMS2-PNA trajectories, since analogous tools for proteins are precisely what makes CG intermediates immediately actionable for downstream MD refinement [103] and drug design applications. Closing this gap will be essential to fully unlock the potential of the integrative framework introduced here.

Together, the present works established a unified CG framework for describing both equilibrium fluctuations and conformational transitions in DNA, RNA, and protein–nucleic acid assemblies. By combining an essential-dynamics refined ENM with BD path sampling, the approach enables efficient exploration of functional motions across a broad range of biological systems and length scales. As cryo-EM, integrative structural biology, and ensemble-based experimental techniques continue to expand the number of available conformational states, computational methods capable of connecting these states through physically plausible transition pathways will become increasingly important. We anticipate that edENM-PNA and eBDIMS2-PNA will provide a practical and scalable framework for investigating the structural mechanisms underlying nucleic acid function and regulation.

## Supplementary Material

gkag807_Supplemental_File

## Data Availability

All codes and data needed to reproduce the results shown in this work are freely available on GitHub at https://github.com/domenicoscaramozzino/Protein_DNA_RNA_edENM. The repository has been linked with Zenodo at https://doi.org/10.5281/zenodo.21331566.
