# Supplementary material for: Modeling conformational transitions in DNA, RNA, and protein–nucleic acid complexes
Source: Nucleic Acids Res. 2026 Aug 26;54(16):gkag807. doi: 10.1093/nar/gkag807 (PMC13507862; doi:10.1093/nar/gkag807)
Supplement: Supplementary file 1 [file gkag807_supplemental_file.pdf]

Supplementary Material

Modeling conformational transitions in DNA, RNA, and protein-nucleic acid complexes

Domenico Scaramozzino[1,†,*], Marco Cannariato[2,†], Byung Ho Lee[1], Marco A. Deriu[2], Laura Orellana[1,*]

[1]*Protein Dynamics and Mutation Lab*, Department of Oncology-Pathology, Karolinska Institute, Solna, 17165, Sweden.
[2]*PolitoBIOMed Lab*, Department of Mechanical and Aerospace Engineering, Politecnico di Torino, Torino, 10129, Italy.

[†]Domenico Scaramozzino and Marco Cannariato contributed equally to this work.

*Corresponding authors: laura.orellana@ki.se; domenico.scaramozzino@ki.se

Supplementary Table 1. Details about the systems and MD simulations used in the study. The references to the publication and to the online data, number of replicas available, simulation length, simulating software, employed force-field, simulation temperature, and training-test split are indicated for each system.

Simulation	Molecular system	# rep	Length [ns]	Software	Force-field	T [K]	Split
Vreede et al. Nucleic Acids Res. 2019[1,2]	DNA	5	200	GROMACS	AMBER03	300	Train
Lemkul Nucleic Acids Res. 2020[3,4]	DNA	3	1000	OpenMM	CHARMM36	298	Train
Lemkul Nucleic Acids Res. 2020[3,4]	DNA	3	1000	OpenMM	CHARMM36	298	Train
Lemkul Nucleic Acids Res. 2020[3,4]	DNA	3	1000	OpenMM	Drude-2017	298	Train
Lemkul Nucleic Acids Res. 2020[3,4]	DNA	3	1000	OpenMM	Drude-2017	298	Test
Kameda et al. Phys. Rev. E 2021[5,6]	DNA	10	50	NAMD	CHARMM36	300	Test
Pyne et al. Nat. Commun. 2021[7,8]	DNA	8	30	AMBER	AMBER99 + parmBSC0 + parmOL4 + parmOL1	300	Test
Salsbury et al. ACS Omega 2022[4,9]	DNA	3	1000	OpenMM	CHARMM36	298	Train
Salsbury et al. ACS Omega 2022[4,9]	DNA	3	1000	OpenMM	CHARMM36	298	Train
Salsbury et al. ACS Omega 2022[4,9]	DNA	3	2000	OpenMM	CHARMM36	298	Train
Salsbury et al. ACS Omega 2022[4,9]	DNA	3	2000	OpenMM	CHARMM36	298	Train
Bochicchio et al. PLoS Comput. Biol. 2018[10,11]	RNA	1	880	GROMACS	AMBERff14SB + OL3	298	Train
Bochicchio et al. PLoS Comput. Biol. 2018[10,11]	RNA	5	880	AMBER	AMBERff14SB + OL3	298	Train
Bochicchio et al. PLoS Comput. Biol. 2018[10,12]	RNA	3	1000	AMBER	AMBERff14SB + OL3 VdW mod	298	Train

Levintov and Vashisth Phys. Chem. Chem. Phys. 2021[13,14]	RNA	14	2000	AMBER	AMBERff99 + OL3	300	Train
De Bisschop et al. Non-coding RNA 2022[15,16]	RNA	1	1000	GROMACS	Amber ff99 + parmBSC0	300	Test
Kameda et al. Front. Mol. Biosci. 2019[17,18]	Complex	10	100	NAMD	CHARMM36	310	Test
Baltrukevich and Bartos Front. Chem. 2023[19,20]	Complex	4	500	AMBER	AMBERff14SB + OL3	310	Train
Baltrukevich and Bartos Front. Chem. 2023[19,20]	Complex	4	500	AMBER	AMBERff19SB + OL3	310	Train
Baltrukevich and Bartos Front. Chem. 2023[19,21]	Complex	4	500	AMBER	AMBERff14SB + OL3	310	Train
Baltrukevich and Bartos Front. Chem. 2023[19,21]	Complex	4	500	AMBER	AMBERff19SB + OL3	310	Train
Baltrukevich and Bartos Front. Chem. 2023[19,20]	Complex	4	500	DESMOND	OPLS4	300	Train
Baltrukevich and Bartos Front. Chem. 2023[19,21]	Complex	4	500	DESMOND	OPLS4	300	Train
Sekulovski et al. Nat. Struct. Mol. Biol. 2023[22,23]	Complex	1	1000	GROMACS	AMBERff14SB + OL3	300	Test
Riccardi et al. PLoS Comput. Biol. 2019[24,25]	Complex	20	50	GROMACS	AMBER03	300	Train
Bochicchio et al. PLoS Comput. Biol. 2018[10,11]	Complex	5	1000	AMBER	AMBERff14SB + OL3	298	Test

Supplementary Table 2. PDB IDs for the multi-model structures forming the experimental dataset for the edENM-NA parametrization. The structures have been divided into training and test sets. Groups of PDB IDs forming a single ensemble are grouped into squared brackets.

Split	PDB
Training set	1A60, 1B36, 1JUA, 1P5O, 2LU0, 2N1Q, 7XH3, 8THV, [8UYK, 8UYL, 8UYM], [7UVT, 7XD3, 7XD4, 7XSK, 7XSL, 7XSM, 7YG8, 7YG9, 7YGA, 7YGB, 7YGC, 7YGD, 8HD6, 8HD7, 8I7N]
Testing set	1AX6, 1BJ2, 1FYP, [1JOX, 1JP0], 2GV3, 2JTP, 2LI4, 2M12, 2M4Q, 2M8Z, 2MQT, 2MTJ, 2N6X, 2PCV, 5N5C, 5UZT, 6H1K, 8S1W, [8UYE, 8UYG, 8UYJ]

Supplementary Table 3. PDB IDs for the NMR structures of protein-nucleic acid complexes used for final parameter refinement and performance testing of edENM-PNA.

PDB
1AHD, 1AUD, 1F6U, 1FJE, 1FNX, 1ULL, 2KEK, 2KM8, 2K1N, 2K7F, 2LT7, 2ME6, 2MF0, 2MKK, 2MRU, 2N3O, 2N82, 2OEH, 6GBM, 6WLH, 7WNR

Supplementary Table 4. PDB IDs for structures in the experimental ensemble dataset for the testing of edENM-PNA. The size of the molecular system is shown by reporting number of C_α atoms (for the protein) and P atoms (for DNA/RNA). Maximum RMSD values in each ensemble as well as the variances associated with PC1 and PC2 are also reported.

Ensemble	Complex	PDB	Number C_α/P atoms	RMSD (Å)	Variance PC1/PC2 (%)*
1	HTH-type transcriptional regulator CueR bound to promoter DNA	4WLS, 4WLW	111/43	5.4	100.0/0.0
2	DinG in complex with ssDNA	6FWR, 6FWS	683/9	4.4	100.0/0.0
3	LigIV catalytic domain with bound DNA	6BKF, 6BKG	561/34	13.5	100.0/0.0
4	Drosha and DGCR8 in complex with Primary MicroRNA	6V5B, 6V5C	1,301/66	11.4	100.0/0.0
5	Ago10-guide-target RNA complex	7SWF, 7SWQ	753/31	5.5	100.0/0.0
6	DNMT5 quaternary complex bound with DNA	7R77, 7R78, 7T02	1,704/16	5.4	99.0/1.0
7	dsRNA-binding protein Staufen homolog 1 bound with dsRNA	6SDW, 6SDY (NMRs)	70/33	4.8	58.3/9.0
8	Flpb recombinase bound with DNA	1FLO, 1M6X, 1P4E	1,685/122	4.1	98.9/1.1

9	Replicative DNA helicase bound with DNA	7T20, 7T22, 7T23	2,661/10	5.6	75.7/24.3
10	DNA topoisomerase 1 bound with DNA	6CQ2, 6CQI, 8CZQ	652/10	3.3	96.8/3.2
11	Group II-encoded protein LTRA bound with Group II-intron RNA	5G2X, 7D1A, 8H2H	426/700	4.7	89.9/10.1
12	50S ribosomal protein L7Ae bound with RNA	5DCV_1, 5DCV_2, 5XTM_1, 5XTM_2, 5Y7M_1, 5Y7M_2	118/46	9.4	67.4/29.9
13	Gag-Pro-Pol polyprotein bound with DNA	5EJK, 7JN3, 7KU7, 7KUI, 8E14	1,158/64	3.3	60.5/35.3
14	DNA primase/helicase bound with DNA	6N7N, 6N7S, 6N7T	1,597/13	7.4	93.0/7.0
15	CRISPR-associated endonuclease C2c1 bound with DNA and RNA	5U30, 5U31, 5U33, 5U34, 5WQE	932/59	3.5	91.6/8.2
16	Rad2p bound with DNA	4Q0Z_1, 4Q0Z_2, 4Q10	588/30	3.9	92.1/7.9
17	DNA polymerase delta bound with DNA	6S1M, 6S1N, 6TNY, 6TNZ	2,399/49	5.5	95.9/4.1
18	CRISPR-associated endonuclease Cas9 bound with RNA	6JDQ, 6JE9, 6KC7, 6KC8, 8HJ4, 8JA0	896/110	16.1	86.2/10.2
19	DNA polymerase gamma 1/2 bound with DNA	4ZTU, 4ZTZ, 5C51, 5C52, 5C53, 8D33, 8D37, 8D3R, 8D42	1,643/41	7.3	68.5/24.1
20	DNA polymerase beta bound with DNA	1HUO, 1HUZ, 2BPF, 2BPG	322/13	6.6	67.8/30.3
21	DNA polymerase beta bound with DNA	3UXP, 3V72, 3V7J, 3V7K	326/14	4.0	92.7/3.8
22	Lactose operon repressor bound with DNA	2KEI, 2KEJ, 2KEK (NMRs)	104/45	5.4	53.1/19.1
23	Human topoisomerase IIbeta in complex with DNA	3QX3, 4G0U, 4G0V, 4G0W, 4J3N, 5GWI, 5GWJ, 5ZAD	1,308/39	5.0	88.1/8.3
24	Replication factor C bound with DNA	7TFH, 7TFI, 7TFJ, 7TIB, 7TID	2,446/38	11.5	98.3/0.9
25	crRNA-guided Csy surveillance complex	6B45, 6B47, 6B48, 6NE0, 6VQV, 7ECV, 7JZW, 7JZX, 7JZY, 7JZZ	2,487/57	12.1	83.3/6.0
26	CRISPR-associated endonuclease Cas9/Csn1 bound with DNA and RNA	4OO8, 4UN3, 4UN4, 4UN5, 4ZT0, 4ZT9, 5B2R, 5B2S, 5B2T, 5FQ5, 5FW1, 5FW2,	1,110/61	14.4	56.6/18.9

	5FW3, 5VW1, 5XBL, 6AEG, 6AI6, 6K3Z, 6K4P, 6K4Q, 6K4S, 6K4U, 6K57, 6O0Z, 6VPC, 7OX7, 7OX8, 7OX9, 7OXA, 7QQO, 7QQP, 7QQQ, 7QQR, 7QQS, 7QQT, 7QQU, 7QQV, 7QQW, 7QQX, 7QQZ, 7QR0, 7QR1, 7QR5, 7QR7, 7QR8, 7S36, 7S38, 7S4X, 7Z4C, 7Z4E, 7Z4H, 7Z4I, 7Z4J, 7Z4K, 7Z4L, 8G1I

*The number of meaningful PCs in an ensemble that contains *n* conformations is *n – 1*, thus ensembles with only 2 conformations have a single PC, which describes the transition vector and captures 100% of the variance of the two-state ensemble. As a result, PC variance values in ensembles with a small number of conformers are not meaningful.

Supplementary Table 5. Set of parameters employed in the random search optimization for the edENM-NA. Numbers in **bold** represent those selected as optimal after refinement.

Bond type	Intra-nucleoside (pyrimidines)	Intra-nucleoside (purines)	Base-base	Backbone	Van der Waals
Distance-dependent decay	No	No	No	Yes	Yes
n	-	-	-	2.7, **2.8**, 2.9, 3.0	**1.0**, 1.1, 1.2, 1.3
C	250, 270, **290**, 310, 330, 350	100, **120**, 140, 160, 180, 200	60, **65**, 70, 75, 80, 85	12, 14, 16, 18, **20**	**25**, 30, 35, 40
Cutoff	-	-	-	9, 10, **11**, 12, 13, 14	9, 10, **11**, 12, 13, 14

Supplementary Table 6. Testing of edENM-NA and comparison against uniform-spring ENM for the testing set of multi-model PDB ensembles in Supplementary Table 2 in terms of overlaps and RMSIP scores between NMs and experimental PCs. Maximum overlaps O_{max} and RMSIP were computed based on the first 3 NMs and 3 PCs. The numbers of the NM and PC vector giving the maximum overlap are indicated in the first and second position of the parenthesis, respectively. The last rows show average and standard deviations for all tested PDB structures as well as *p*-values assessing the statistical significance of the differences.

**PDB**	$\mathbf{O_{max}^{ENM}}$	$\mathbf{O_{max}^{edENM-NA}}$	$\mathbf{RMSIP^{ENM}}$	$\mathbf{RMSIP^{edENM-NA}}$
1AX6	$0.583^{(3,1)}$	$0.738^{(1,1)}$	0.528	0.553
1BJ2	$0.713^{(1,2)}$	$0.621^{(1,2)}$	0.690	0.653
1FYP	$0.769^{(1,1)}$	$0.744^{(2,2)}$	0.772	0.893
1JOX	$0.728^{(1,2)}$	$0.720^{(3,1)}$	0.704	0.634
1JP0	$0.913^{(1,1)}$	$0.736^{(1,1)}$	0.657	0.670
2GV3	$0.576^{(2,1)}$	$0.600^{(1,3)}$	0.532	0.564
2JTP	$0.566^{(2,1)}$	$0.658^{(1,1)}$	0.557	0.679
2LI4	$0.852^{(2,2)}$	$0.892^{(2,2)}$	0.911	0.914
2M12	$0.745^{(3,2)}$	$0.607^{(2,2)}$	0.604	0.618
2M4Q	$0.783^{(2,1)}$	$0.819^{(1,1)}$	0.707	0.721
2M8Z	$0.797^{(1,1)}$	$0.769^{(1,1)}$	0.810	0.806
2MQT	$0.628^{(2,1)}$	$0.797^{(2,1)}$	0.530	0.695
2MTJ	$0.852^{(2,1)}$	$0.737^{(1,1)}$	0.594	0.675
2N6X	$0.904^{(1,1)}$	$0.907^{(1,1)}$	0.793	0.808
2PCV	$0.636^{(1,1)}$	$0.930^{(1,1)}$	0.570	0.758
5N5C	$0.950^{(1,1)}$	$0.926^{(1,1)}$	0.609	0.628
5UZT	$0.603^{(1,1)}$	$0.659^{(2,1)}$	0.613	0.621
6H1K	$0.779^{(1,2)}$	$0.723^{(1,2)}$	0.616	0.687
8S1W	$0.775^{(1,1)}$	$0.726^{(1,1)}$	0.707	0.712
8UYE	$0.785^{(3,1)}$	$0.805^{(2,1)}$	0.515	0.661
8UYG	$0.798^{(1,1)}$	$0.858^{(1,1)}$	0.632	0.666
8UYJ	$0.804^{(1,1)}$	$0.858^{(1,1)}$	0.581	0.691
All	0.752 ± 0.110	0.765 ± 0.099	0.647 ± 0.102	0.696 ± 0.090
Wilcoxon one-sided p-value (edENM-NA > ENM)	0.3278		0.0004	

Supplementary Table 7. Set of parameters for the random search optimization of protein-nucleic acid interactions for edENM-PNA. Numbers in **bold** represent those selected as optimal after refinement.

Bond type	Type-1	Type-2
Distance-dependent decay	Yes	Yes
n	1.9, 2.0, 2.1, **2.2**	**1.0**
C	15, 20, **25**	**30**, 35, 40
Cutoff	**8**, 9	**8**, 9

Supplementary Table 8. Testing of edENM-PNA and comparison against uniform-spring ENM and edProt for the set of NMR models in Supplementary Table 3 in terms of overlap and RMSIP scores between NMs and experimental PCs. Maximum overlaps O_{max} and RMSIP were computed based on the first 3 NMs and 3 PCs. The numbers of the NM and PC vector giving the maximum overlap are indicated in the first and second position of the parenthesis, respectively. The last row shows average and standard deviations for all tested PDB structures.

PDB	O_{max}^{ENM}	O_{max}^{edProt}	$O_{max}^{edENM-PNA}$	$RMSIP_3^{ENM}$	$RMSIP_3^{edProt}$	$RMSIP_3^{edENM-PNA}$
1AHD	$0.551^{(2,2)}$	$0.457^{(2,2)}$	$0.408^{(2,1)}$	0.415	0.425	0.393
1AUD	$0.717^{(1,2)}$	$0.714^{(1,2)}$	$0.622^{(1,1)}$	0.674	0.659	0.555
1F6U	$0.419^{(2,2)}$	$0.492^{(3,3)}$	$0.628^{(2,3)}$	0.300	0.561	0.567
1FJE	$0.656^{(3,1)}$	$0.603^{(3,1)}$	$0.842^{(1,1)}$	0.535	0.591	0.760
1FNX	$0.332^{(1,2)}$	$0.682^{(3,1)}$	$0.752^{(3,1)}$	0.282	0.514	0.480
1ULL	$0.544^{(1,1)}$	$0.542^{(1,1)}$	$0.620^{(1,1)}$	0.536	0.538	0.420
2KEK	$0.555^{(3,1)}$	$0.549^{(3,3)}$	$0.607^{(1,1)}$	0.569	0.582	0.599
2KM8	$0.739^{(1,1)}$	$0.876^{(1,1)}$	$0.665^{(1,1)}$	0.535	0.586	0.550
2K1N	$0.251^{(2,2)}$	$0.369^{(3,2)}$	$0.447^{(2,2)}$	0.204	0.320	0.366
2K7F	$0.423^{(2,1)}$	$0.452^{(2,1)}$	$0.619^{(1,2)}$	0.320	0.337	0.522
2LT7	$0.778^{(2,1)}$	$0.791^{(1,1)}$	$0.664^{(1,1)}$	0.496	0.511	0.516
2ME6	$0.211^{(3,2)}$	$0.292^{(3,2)}$	$0.343^{(1,1)}$	0.194	0.269	0.278
2MF0	$0.807^{(2,1)}$	$0.794^{(1,1)}$	$0.752^{(1,1)}$	0.552	0.559	0.563
2MKK	$0.503^{(2,1)}$	$0.459^{(3,1)}$	$0.689^{(1,1)}$	0.579	0.536	0.570
2MRU	$0.378^{(2,1)}$	$0.345^{(2,1)}$	$0.513^{(2,1)}$	0.309	0.346	0.434
2N30	$0.560^{(1,2)}$	$0.561^{(1,2)}$	$0.790^{(2,1)}$	0.608	0.610	0.685
2N82	$0.492^{(3,3)}$	$0.515^{(1,1)}$	$0.359^{(1,1)}$	0.530	0.425	0.370
2OEH	$0.216^{(3,2)}$	$0.339^{(3,3)}$	$0.322^{(2,3)}$	0.217	0.344	0.359
6GBM	$0.659^{(1,2)}$	$0.722^{(2,1)}$	$0.767^{(1,1)}$	0.629	0.723	0.648
6WLH	$0.632^{(2,2)}$	$0.562^{(2,1)}$	$0.486^{(1,2)}$	0.626	0.533	0.486
7WNR	$0.385^{(2,2)}$	$0.406^{(2,2)}$	$0.482^{(3,2)}$	0.385	0.406	0.482
All	0.515 ± 0.175	0.550 ± 0.159	0.590 ± 0.151	0.452 ± 0.152	0.494 ± 0.120	0.505 ± 0.115

Supplementary Table 9. *P*-value statistical test (Wilcoxon one-sided) between edENM-PNA, ENM, and edProt for O_{max} values (left) and RMSIP scores (right) obtained from the dataset of protein-nucleic acid NMR structures from Supplementary Table 8. The matrix shows one-sided *p*-values with the hypothesis that the model in the column provides values significantly higher than the model in the row.

**Model**	ENM	edProt	edENM-PNA	ENM	edProt	edENM-PNA
ENM	-	0.0895	0.0210	-	0.0097	0.0273
edProt	0.9161	-	0.0786	0.9912	-	0.3414
edENM-PNA	0.9808	0.9265	-	0.9749	0.6711	-

Supplementary Table 10. Testing of edENM-PNA and comparison against ENM and edProt for the testing set of ensembles in Supplementary Table 3 in terms of overlaps and RMSIP scores between NMs and experimental PCs. O_{max} and RMSIP scores were computed based on the first 3 NMs and the maximum between 3 and the number of available experimental PCs. Each row reports average (left) and maximum (right) scores considering individual computations for each PDB in the ensemble. The last two rows report average scores and standard deviations for all tested ensembles considering aggregated scores by average or maximum values.

Ensemble	O_{max}^{ENM}	O_{max}^{edProt}	$O_{max}^{edENM-PNA}$	$RMSIP^{ENM}$	$RMSIP^{edProt}$	$RMSIP^{edENM-PNA}$
1	0.366/0.367	0.390/0.491	0.634/0.702	0.271/0.367	0.076/0.143	0.191/0.203
2	0.634/0.684	0.635/0.684	0.622/0.669	0.634/0.684	0.635/0.684	0.622/0.669
3	0.406/0.500	0.327/0.390	0.396/0.502	0.105/0.165	0.227/0.390	0.323/0.502
4	0.582/0.604	0.577/0.613	0.568/0.603	0.141/0.195	0.287/0.541	0.282/0.533
5	0.278/0.317	0.254/0.323	0.244/0.312	0.238/0.317	0.254/0.323	0.125/0.177
6	0.340/0.401	0.376/0.453	0.375/0.439	0.213/0.303	0.263/0.297	0.280/0.323
7	0.619/0.705	0.640/0.740	0.579/0.825	0.581/0.670	0.621/0.728	0.506/0.638
8	0.680/0.730	0.539/0.554	0.552/0.566	0.570/0.583	0.497/0.512	0.483/0.494
9	0.459/0.485	0.448/0.456	0.444/0.455	0.462/0.497	0.466/0.500	0.465/0.499
10	0.597/0.600	0.619/0.625	0.619/0.629	0.441/0.445	0.483/0.488	0.489/0.493
11	0.098/0.120	0.081/0.094	0.088/0.095	0.074/0.086	0.065/0.075	0.065/0.085
12	0.502/0.584	0.515/0.585	0.565/0.621	0.462/0.562	0.470/0.562	0.476/0.576
13	0.421/0.427	0.445/0.468	0.437/0.490	0.445/0.471	0.510/0.523	0.410/0.538
14	0.447/0.532	0.379/0.422	0.368/0.426	0.447/0.481	0.401/0.445	0.395/0.429
15	0.558/0.680	0.574/0.736	0.571/0.730	0.409/0.477	0.433/0.495	0.437/0.499
16	0.900/0.967	0.764/0.781	0.877/0.908	0.765/0.779	0.753/0.789	0.783/0.788
17	0.888/0.937	0.616/0.650	0.772/0.875	0.595/0.615	0.582/0.587	0.575/0.583
18	0.368/0.720	0.592/0.780	0.682/0.812	0.271/0.498	0.494/0.677	0.637/0.739
19	0.286/0.659	0.503/0.564	0.553/0.605	0.254/0.506	0.445/0.505	0.534/0.547
20	0.733/0.814	0.830/0.877	0.837/0.891	0.618/0.644	0.637/0.643	0.634/0.642
21	0.504/0.525	0.527/0.566	0.546/0.575	0.461/0.553	0.505/0.562	0.605/0.641
22	0.664/0.792	0.704/0.795	0.722/0.815	0.697/0.783	0.674/0.742	0.757/0.794
23	0.473/0.535	0.449/467	0.425/0.621	0.424/0.469	0.400/0.420	0.391/0.527
24	0.400/0.576	0.421/0.767	0.387/0.580	0.325/0.406	0.336/0.500	0.321/0.527
25	0.315/0.468	0.277/0.358	0.312/0.386	0.265/0.436	0.258/0.297	0.277/0.305
26	0.326/0.612	0.451/0.657	0.486/0.678	0.303/0.584	0.424/0.544	0.454/0.565
All (avg.)	0.494 ± 0.185	0.497 ± 0.162	0.525 ± 0.177	0.403 ± 0.181	0.431 ± 0.170	0.443 ± 0.176
All (max.)	0.590 ± 0.184	0.573 ± 0.176	0.608 ± 0.188	0.484 ± 0.170	0.499 ± 0.169	0.512 ± 0.172

Supplementary Table 11. *P*-value statistical test (Wilcoxon one-sided) between edENM-PNA, ENM, and edProt for O_{max} values (left) and RMSIP scores (right) obtained from the dataset of protein-nucleic acid ensembles, whose aggregated values are reported in Supplementary Table 10. These *p*-value results have been obtained considering comparisons between similarity scores at the level of each individual PDB. The matrix shows one-sided *p*-values with the hypothesis that the model in the column provides values significantly higher than the model in the row.

**Model**	ENM	edProt	edENM-PNA	ENM	edProt	edENM-PNA
ENM	-	$< 1 \times 10^{-6}$	$< 1 \times 10^{-6}$	-	$< 1 \times 10^{-6}$	$< 1 \times 10^{-6}$
edProt	1.000	-	0.00008	1.000	-	0.0001
edENM-PNA	1.000	0.9999	-	1.000	0.9999	-

Supplementary Table 12. *P*-value statistical test (Wilcoxon one-sided) between edENM-PNA, ENM, and edProt for O_{max} values (left) and RMSIP scores (right) obtained from the dataset of protein-nucleic acid ensembles, whose aggregated values are reported in Supplementary Table 10. These *p*-value results have been obtained considering comparisons between similarity scores aggregated through average values at the level of each ensemble. The matrix shows one-sided *p*-values with the hypothesis that the model in the column provides values significantly higher than the model in the row.

**Model**	ENM	edProt	edENM-PNA	ENM	edProt	edENM-PNA
ENM	-	0.34	0.23	-	0.05	0.12
edProt	0.66	-	0.04	0.95	-	0.20
edENM-PNA	0.77	0.96	-	0.88	0.80	-

Supplementary Table 13. *P*-value statistical test (Wilcoxon one-sided) between edENM-PNA, ENM, and edProt for O_{max} values (left) and RMSIP scores (right) obtained from the dataset of protein-nucleic acid ensembles, whose aggregated values are reported in Supplementary Table 10. These *p*-value results have been obtained considering comparisons between similarity scores aggregated through maximum values at the level of each ensemble. The matrix shows one-sided *p*-values with the hypothesis that the model in the column provides values significantly higher than the model in the row.

**Model**	ENM	edProt	edENM-PNA	ENM	edProt	edENM-PNA
ENM	-	0.56	0.18	-	0.35	0.19
edProt	0.42	-	0.003	0.65	-	0.04
edENM-PNA	0.82	0.997	-	0.81	0.96	-

Supplementary Table 14. *P*-value statistical test (Wilcoxon one-sided) between edENM-PNA, ENM, and edProt for NM1 collectivity values obtained from the dataset of protein-nucleic acid ensembles, whose aggregated values are reported in Supplementary Table 10. These *p*-value results have been obtained considering comparisons between similarity scores at the level of each individual PDB. The matrix shows one-sided *p*-values with the hypothesis that the model in the column provides values significantly higher than the model in the row.

**Model**	ENM	edProt	edENM-PNA
ENM	-	$< 1 \times 10^{-6}$	$< 1 \times 10^{-6}$
edProt	1.000	-	0.00007
edENM-PNA	1.000	0.9999	-

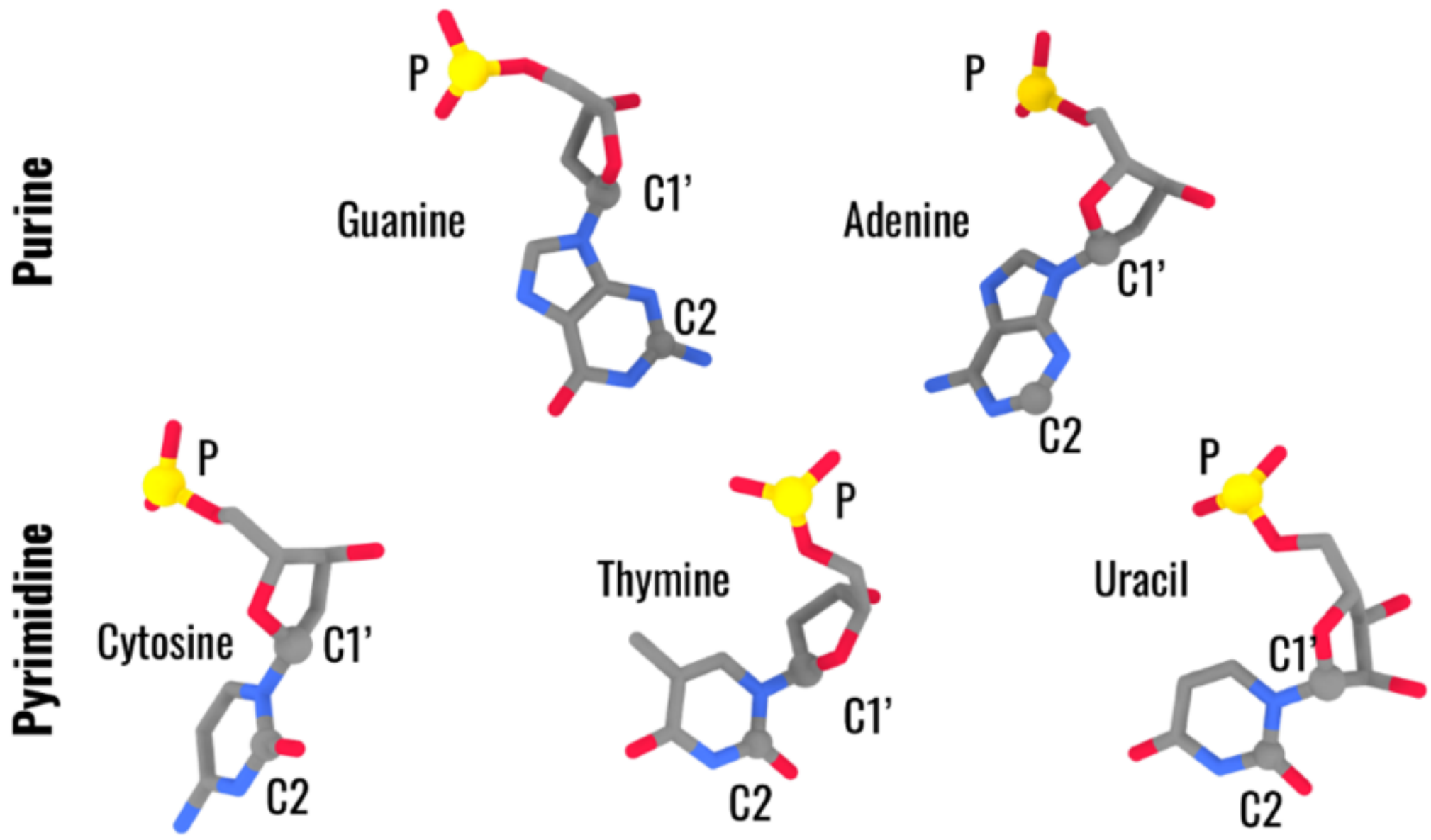

Supplementary Figure 1. Representation of the three-bead SBP model from Pinamonti et al.[26] for the five nucleotide types (G, A, C, T, U). The three beads of the ENM, shown as spheres, are located in the geometrical positions of P, C1′, and C2 atoms.

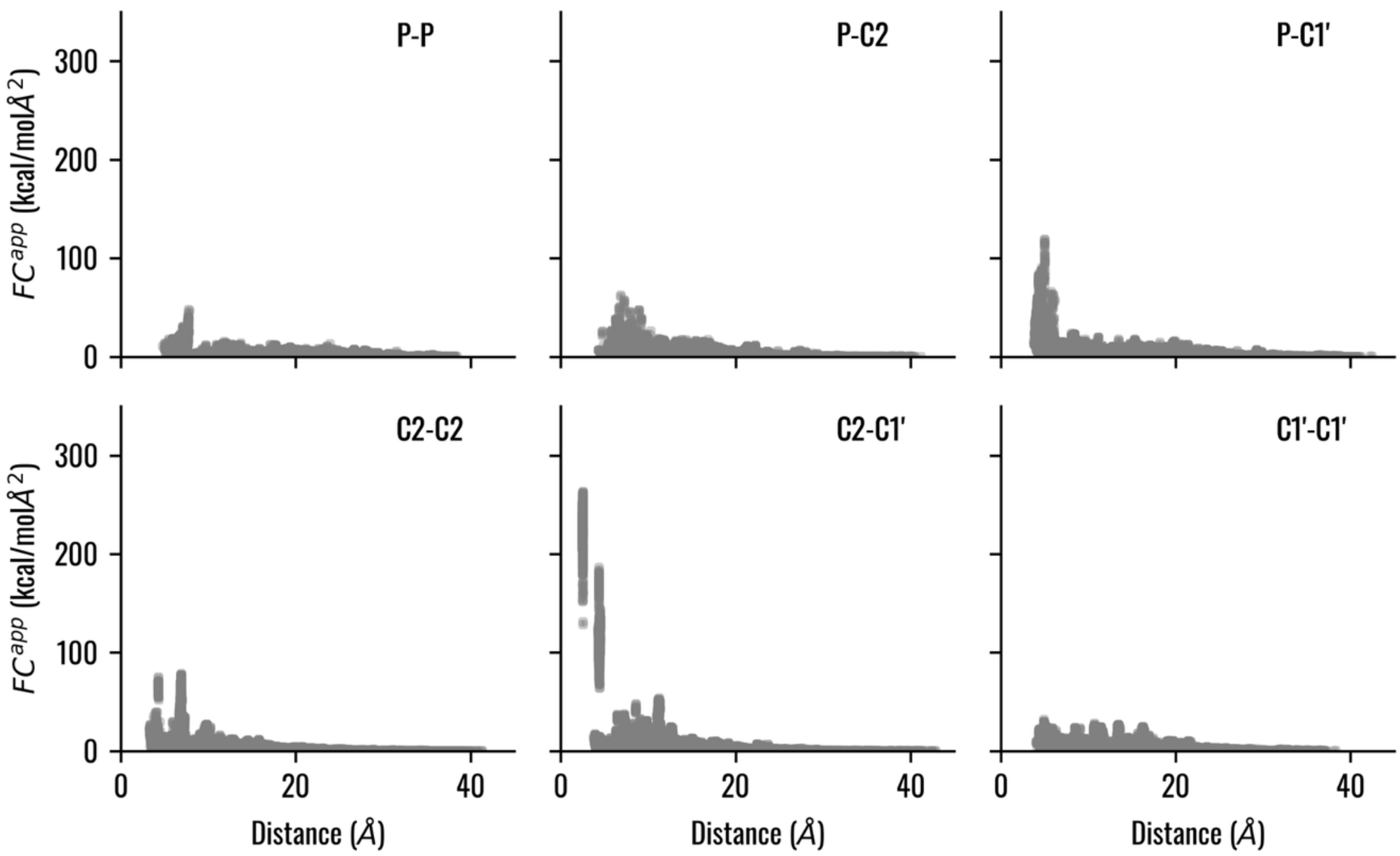

Supplementary Figure 2. Distribution of the apparent force constants FC_{ij}^{app} as a function of the average distance d_{ij}^{a} in the windows of the training MD dataset of DNA-only systems. The apparent force constants between the different interacting couples between P, C1′ and C2 beads are shown.

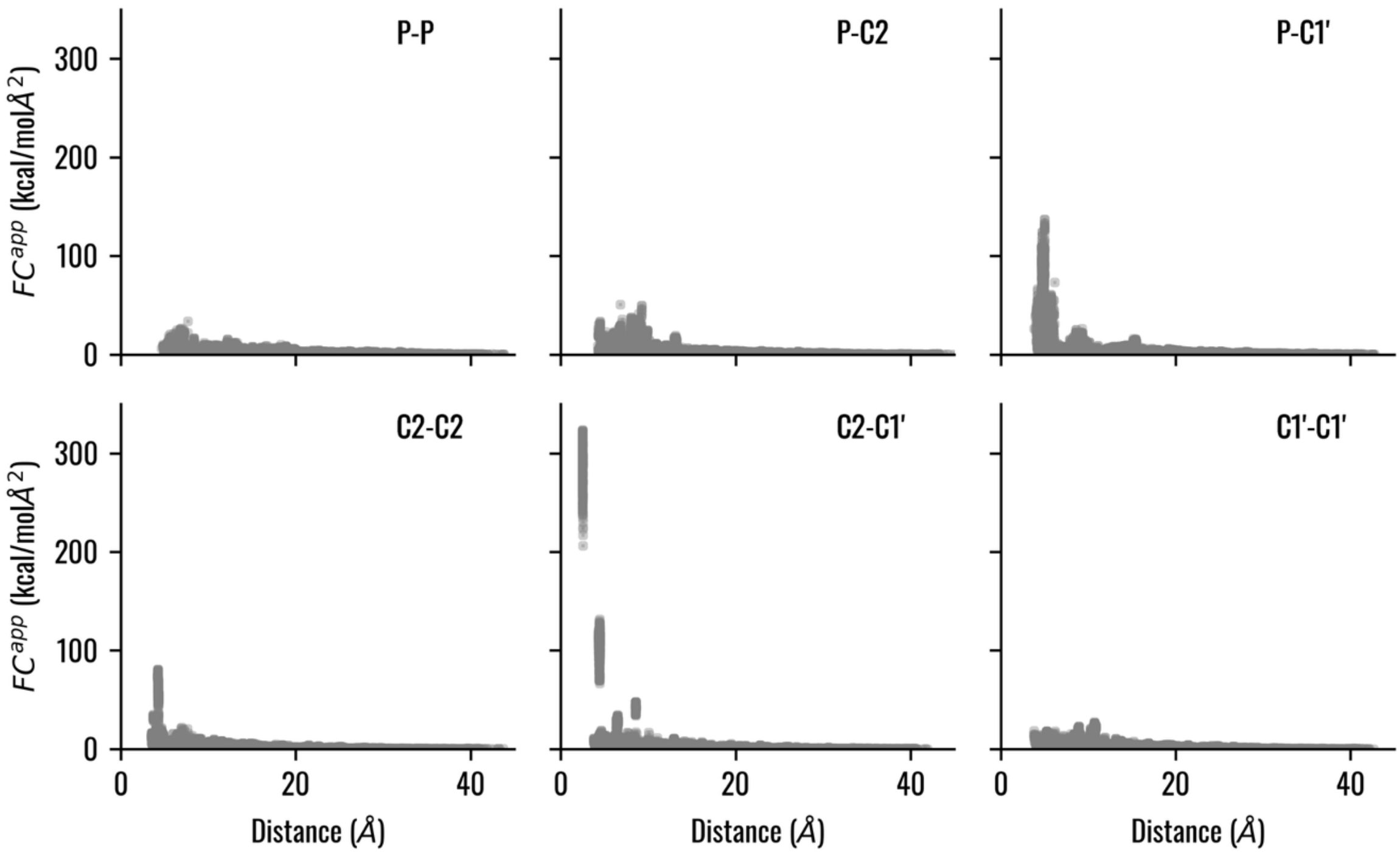

Supplementary Figure 3. Distribution of the apparent force constants FC_{ij}^{app} as a function of the average distance d_{ij}^{a} in the windows of the training MD dataset of RNA-only systems. The apparent force constants between the different interacting couples between P, C1′ and C2 beads are shown.

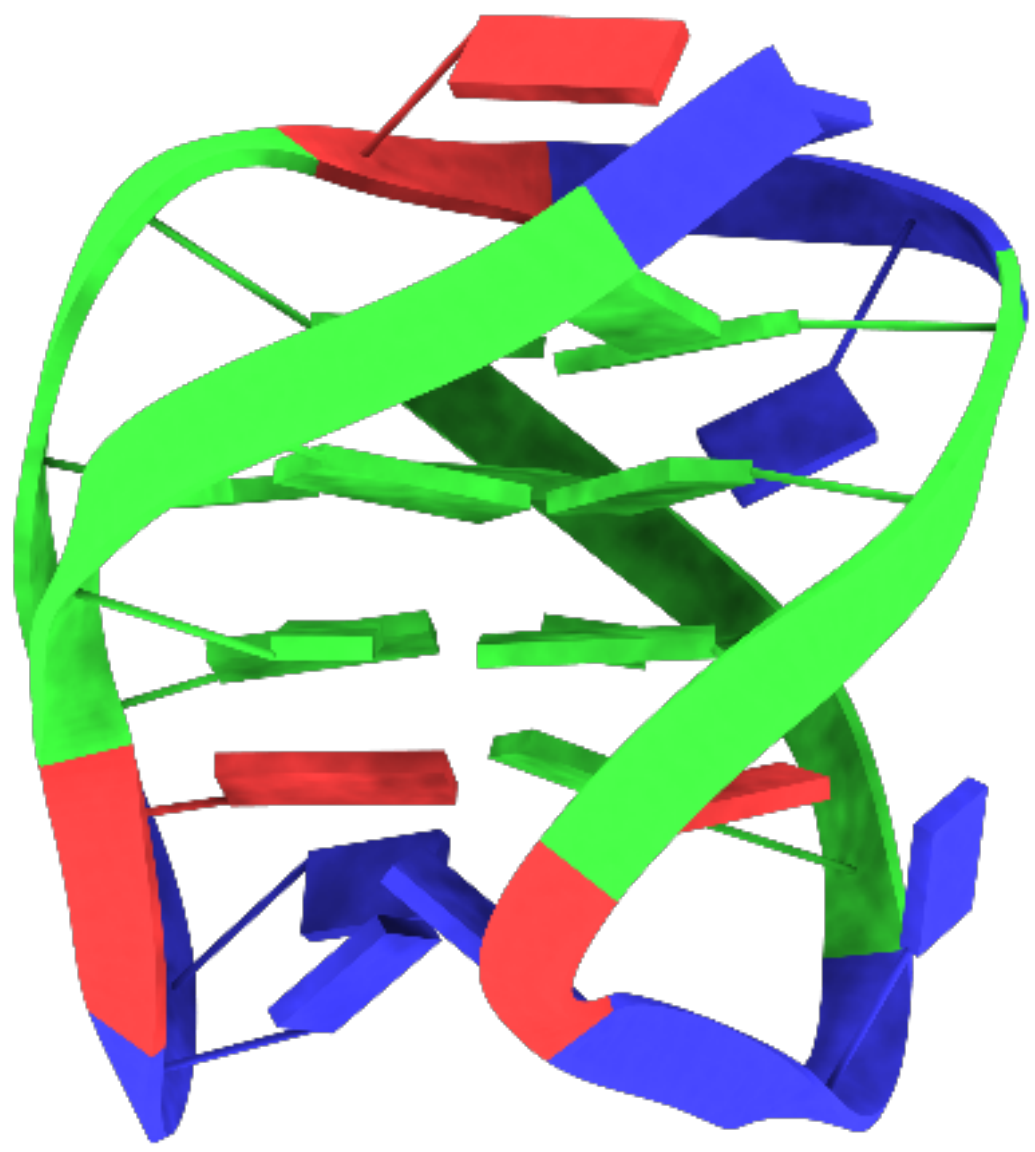

Supplementary Figure 4. Representation of a DNA β-hairpin with strong GG stacking interactions (guanines in green).

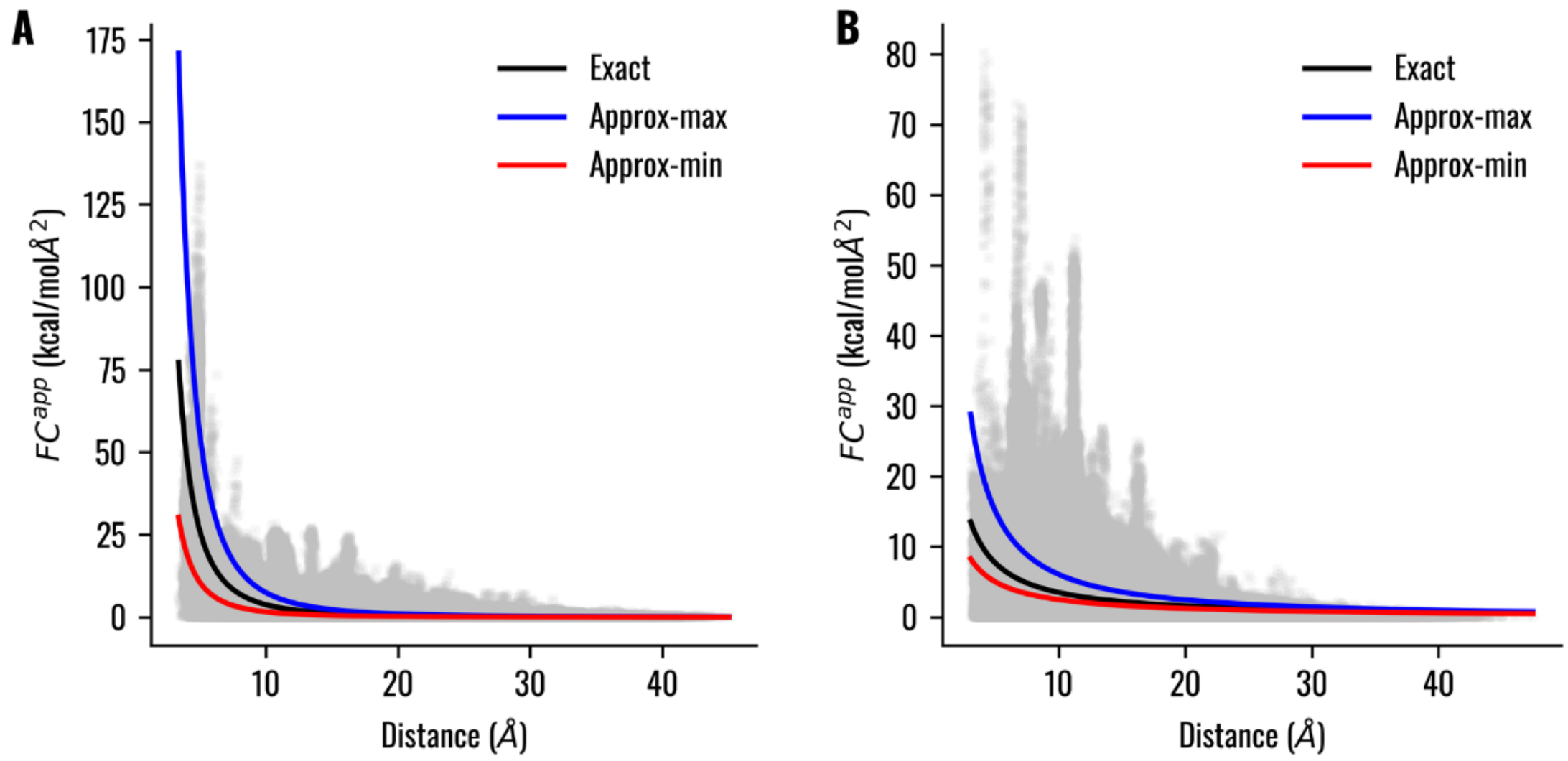

Supplementary Figure 5. Fitting intervals for the (A) "*backbone*" and (B) "*Van der Waals*" interaction types in the edENM-NA. In the apparent force constant versus distance plot, the black line represents the function obtained by the fitting procedure, while the red and blue lines represent, respectively, the curves associated with minimum and maximum values of the parameters used in the random search optimization.

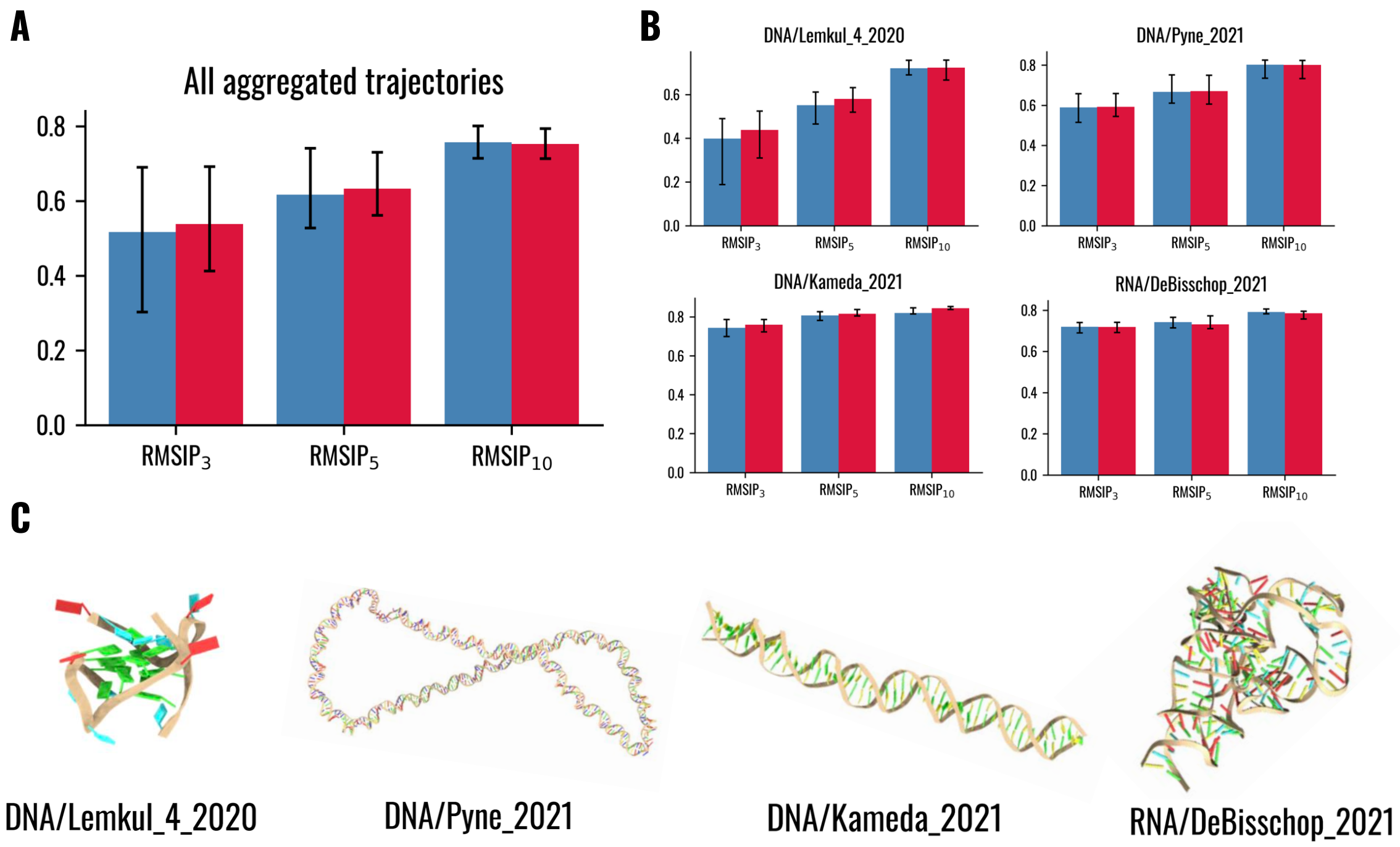

Supplementary Figure 6. Comparison between the uniform-spring ENM from Pinamonti et al.[26] (blue) and the edENM-NA (red) in terms of agreement with the conformational dynamics observed in the four test MD simulations for DNA- and RNA-only systems. (A) Average RMSIP values ($RMSIP_3$, $RMSIP_5$, $RMSIP_{10}$) aggregated for the four testing MD simulations. Error bars refer to the first and third quantiles across the different MD trajectory windows. (B) Comparison with individual MD simulations from Lemkul[3], Pyne et al.[7], Kameda et al.[5], and De Bisschop et al.[15]. A graphical representation of each nucleic acid system is shown in (C).

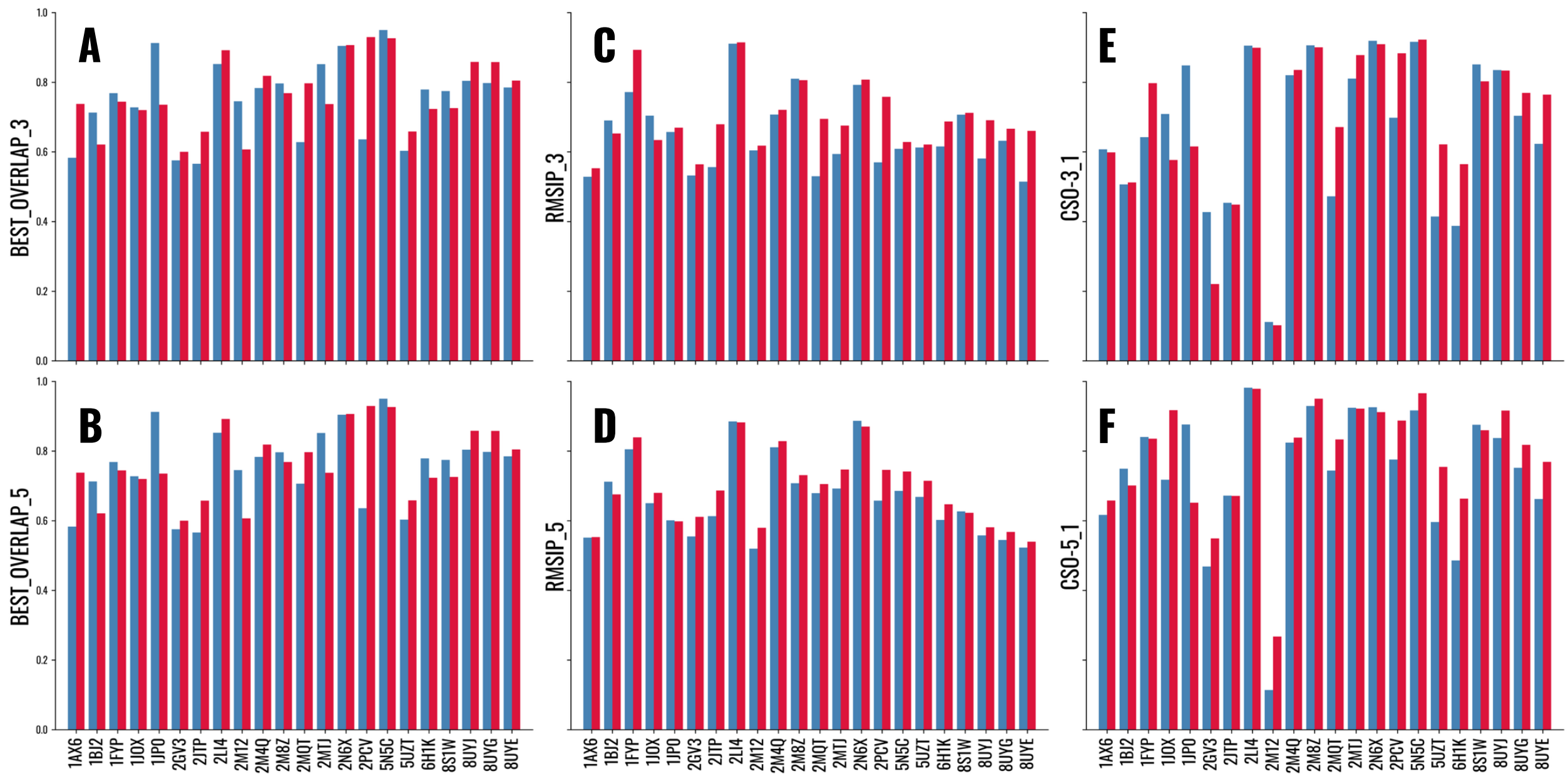

Supplementary Figure 7. Comparison between the uniform-spring ENM from Pinamonti et al.[26] (blue) and the edENM-NA (red) and the experimental PCs from the test set of multi-model ensembles of DNA- and RNA-only systems from Supplementary Table 2: (A) O_{max} for the first 3 sets of NM-PC modes; (B) O_{max} for the first 5 sets of modes; (C) RMSIP for the first 3 sets of modes; (D) RMSIP for the first 5 sets of modes; (E) Cumulative Squared Overlap (CSO) for the first 3 NMs to cover PC1; (F) CSO for the first 5 NMs to cover PC1.

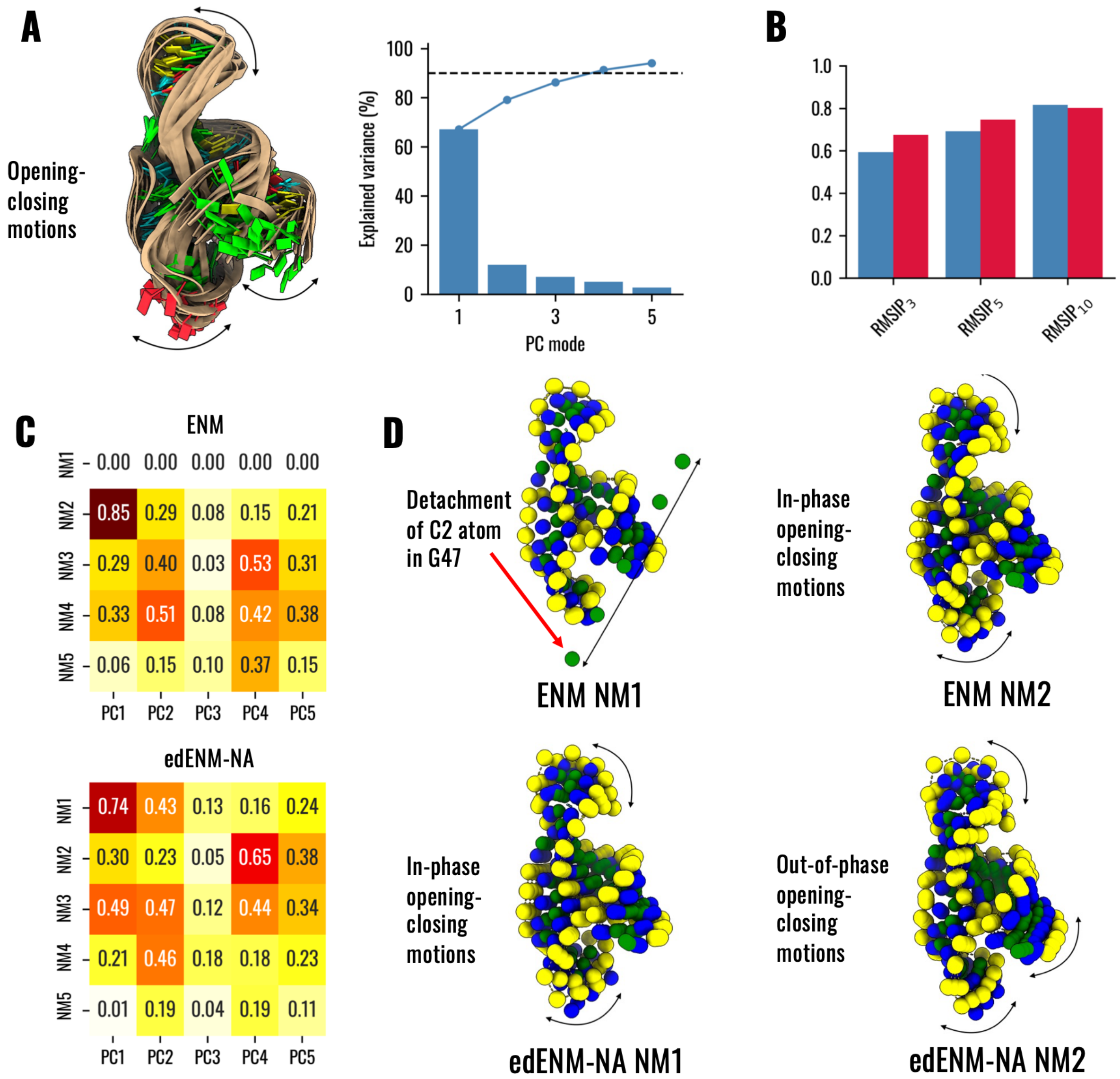

Supplementary Figure 8. Comparison between ENM, edENM-NA and PC modes from the NMR ensemble (PDB: 2MTJ) of the III-IV-V three-way junction of VS ribozyme. (A) Ensemble of 21 models in the solution NMR ensemble used for PCA. The black arrows highlight the opening-closing motions apparent from the experimental ensemble, which dominates PC1. The graph shows the variance captured by each PC mode (blue bars), the cumulative variance of the first PC modes (continuous blue line), and a threshold of 90% variance (dashed black line). PC1 captures ~67% variance and PC1+PC2 together allow to capture ~80% variance in the ensemble. (B) Comparison between ENM (blue bars) and edENM-NA (red) in terms of RMSIP scores (considering both 3, 5, and 10 modes) between NMs and experimental PCs. (C) Matrix of overlaps between the first 5 PC modes and the first 5 NMs from ENM and edENM-NA. Maximum overlaps are found between NM2 and PC1 for ENM (0.85 overlap) and between NM1 and PC1 for edENM-NA (0.74). Note how NM1 of ENM is completely uncorrelated to experimental PCs from the NMR ensemble. (D) Graphical representation of the motions associated to NM1-NM2 for ENM (above) and edENM-NA (below). Beads represent P (yellow), C1′ (blue) and C2 (green) positions. Note how both models allow to capture the opening-closing motions observed in the NMR ensemble, with NM2 of ENM with a ~0.11 better overlap than NM1 of edENM-NA. However, the main mode of ENM (NM1) exhibits a detachment of the C2 bead in the terminal nucleotide G47, which makes NM1 completely uncorrelated to the experimental motion. This detachment is clearly prevented in the edENM-NA due to stronger short-range interactions compared to the uniform-spring ENM.

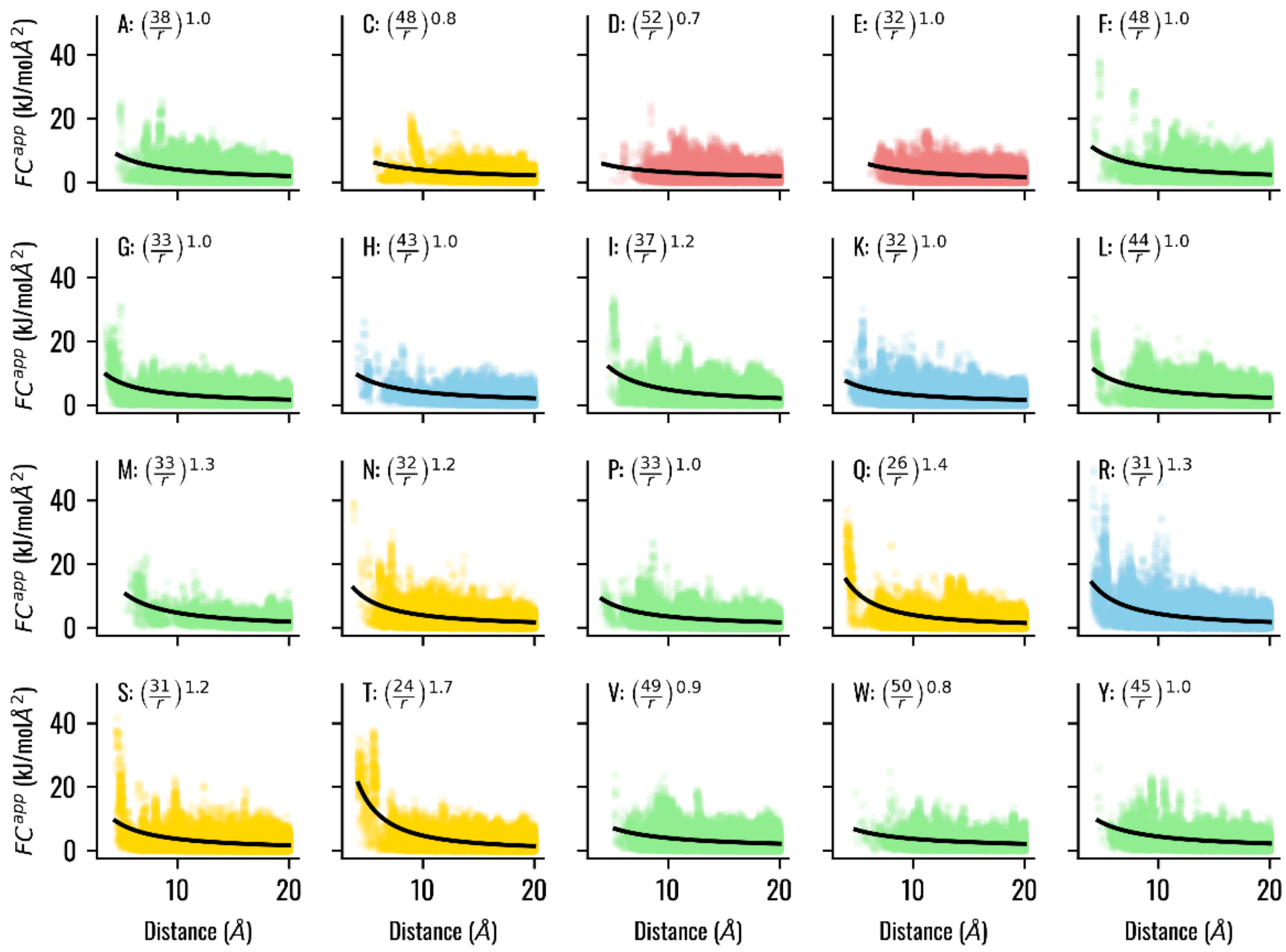

Supplementary Figure 9. Distribution of the C_α-P apparent force constants FC_{ij}^{app} as a function of the average distance d_{ij}^{a} in the window in the training MD dataset of the protein-nucleic acid systems. The apparent force constants with the different amino acids are shown and the points are colored according to the amino acid type, with acidic, basic, polar, and hydrophobic residues highlighted in red, blue, yellow, and green, respectively. Each point is semi-transparent, so darker regions correspond to higher density of data. The black line indicates the function that best fits the data, whose mathematical expression is reported in the plot.

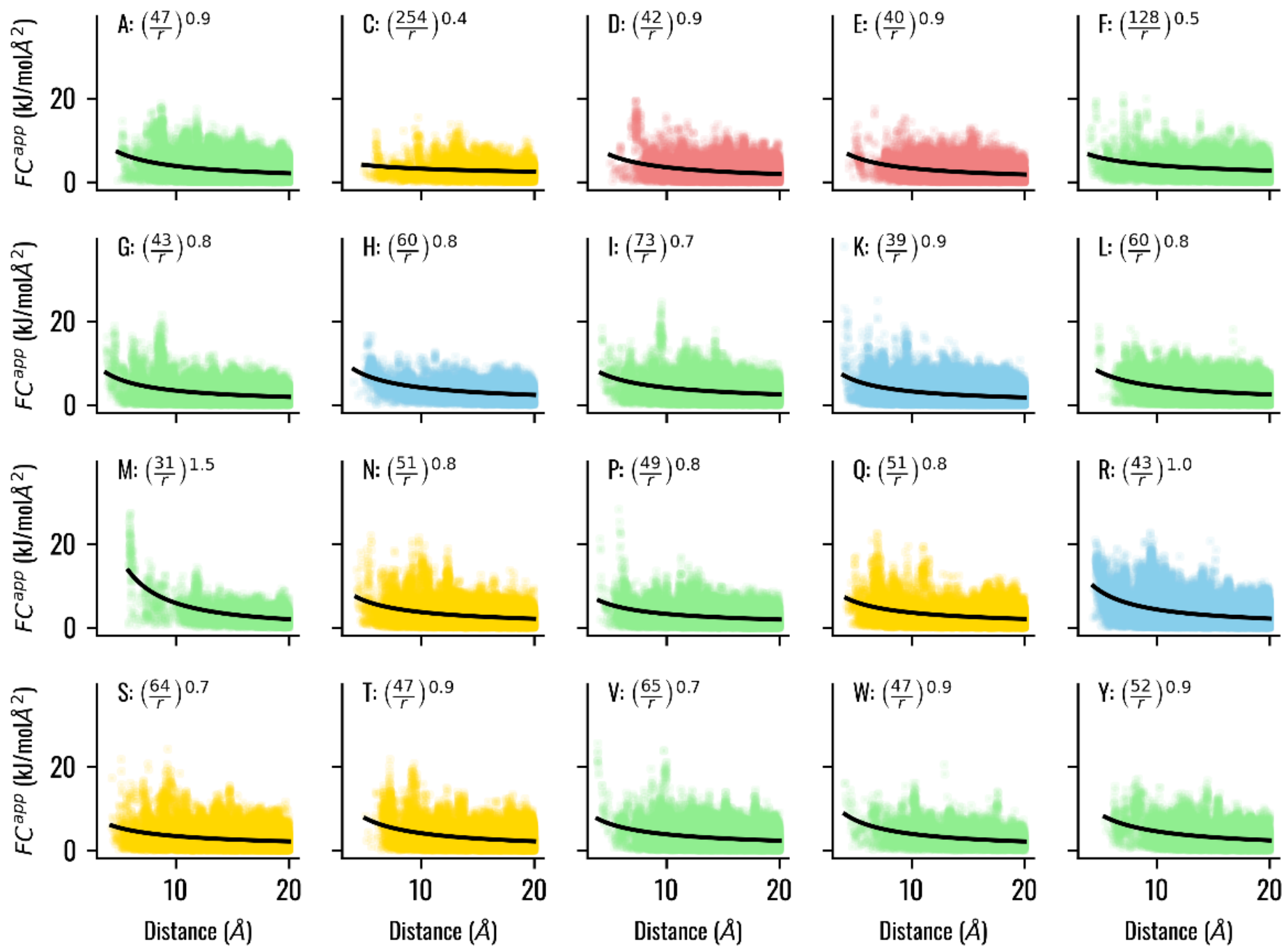

Supplementary Figure 10. Distribution of the C_α-C1′ apparent force constants FC_{ij}^{app} as a function of the average distance d_{ij}^{a} in the window in the training MD dataset of the protein-nucleic acid systems. The apparent force constants with the different amino acids are shown and the points are colored according to the amino acid type, with acidic, basic, polar, and hydrophobic residues highlighted in red, blue, yellow, and green, respectively. Each point is semi-transparent, so darker regions correspond to higher density of data. The black line indicates the function that best fits the data, whose mathematical expression is reported in the plot.

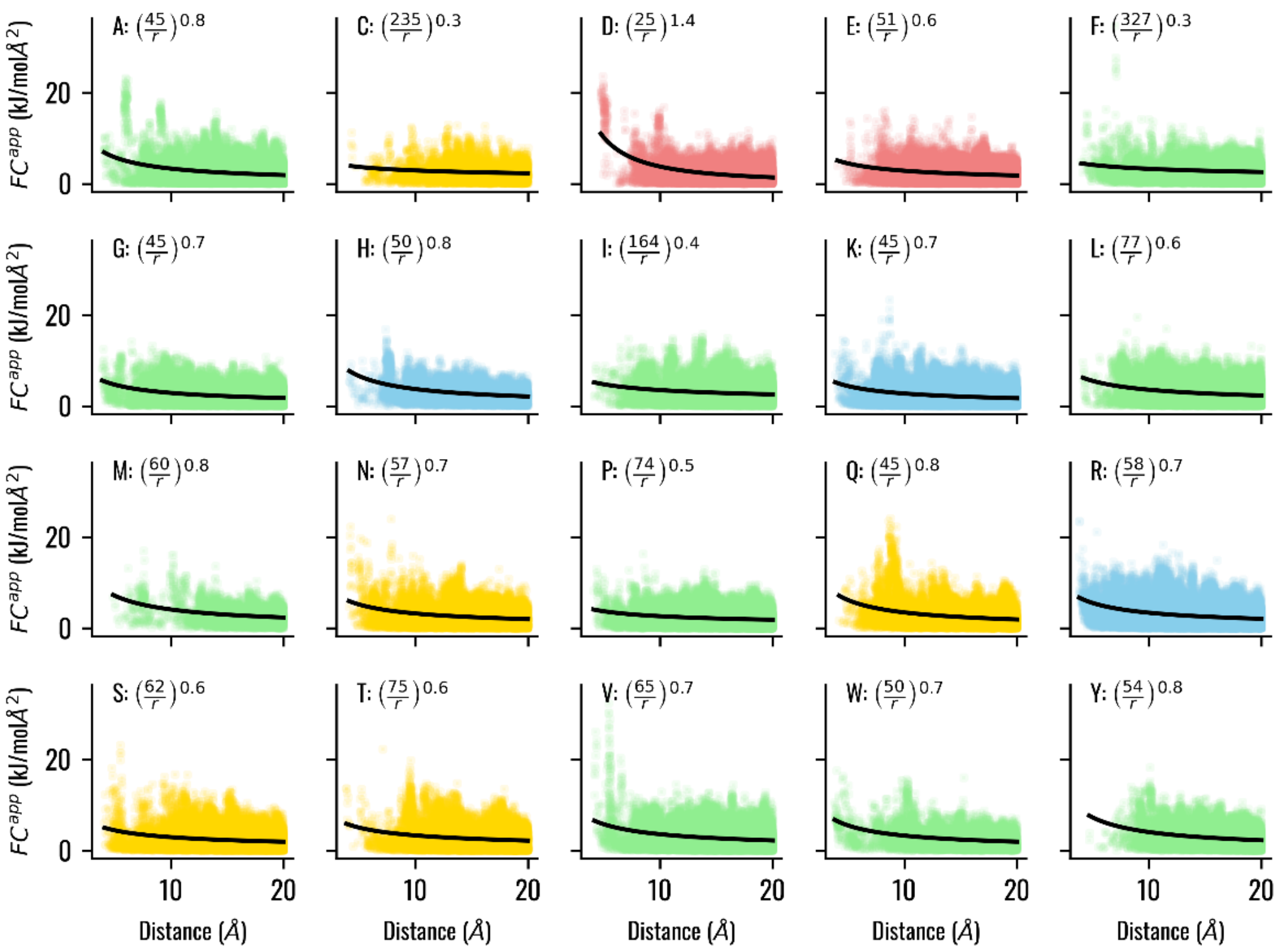

Supplementary Figure 11. Distribution of the C$_\alpha$-C2 apparent force constants FC_{ij}^{app} as a function of the average distance d_{ij}^{a} in the window in the training MD dataset of the protein-nucleic acid systems. The apparent force constants with the different amino acids are shown and the points are colored according to the amino acid type, with acidic, basic, polar, and hydrophobic residues highlighted in red, blue, yellow, and green, respectively. Each point is semi-transparent, so darker regions correspond to higher density of data. The black line indicates the function that best fits the data, whose mathematical expression is reported in the plot.

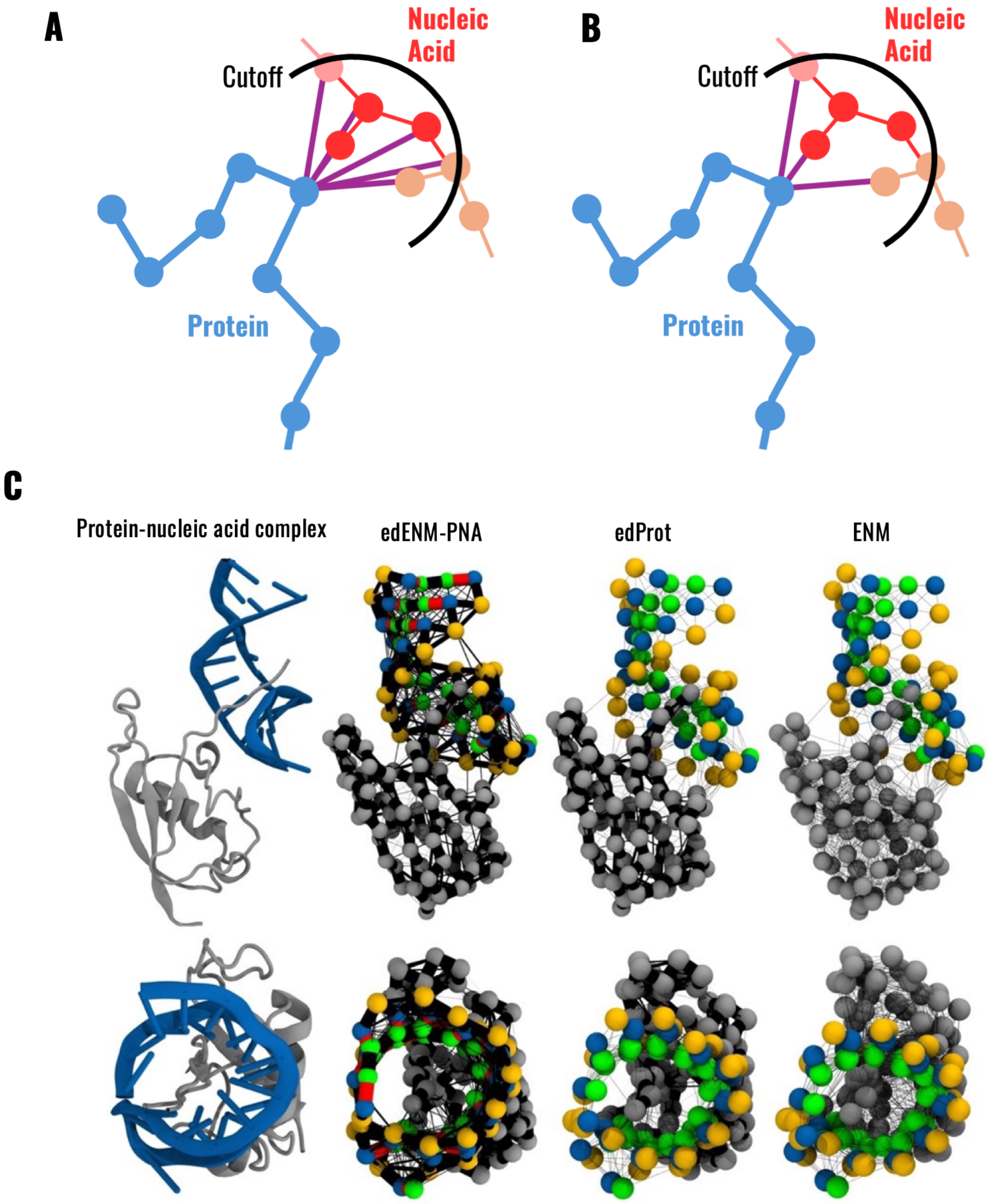

Supplementary Figure 12. (A,B): Representation of the (A) standard and (B) proposed approach to define the protein-nucleic acid contacts at the interface between the protein and the nucleic acid. In the standard approach, each protein bead is connected to all nucleic acid beads within a spatial cutoff, while in the proposed approach protein beads are only connected to one bead per nucleic acid residue, i.e., the bead that is closest to the protein C_α atom. In the figure, protein residues are shown as blue beads, and different residues in the nucleic acid with different colors (pink, red, orange), and protein-nucleic acid contacts as purple lines. The black spherical curve represents the space delimited by the spatial cutoff centered on each C_α atom. (C) Representation of different protein-nucleic acid network models for human SF3A1 ubiquitin-like domain in complex with U1 snRNA stem-loop 4 (PDB ID: 7P0V). The upper panel shows a lateral view, the lower panel a view from above. edENM-PNA represents our optimized network model for the whole protein-nucleic acid complex. edProt uses the edENM parametrization for the protein, but the standard ENM from Pinamonti et al.[26] for the nucleic acid. ENM uses uniform-spring ENM topologies for both. Bead colors and spring diameters are equivalent to those from Fig. 4 in the main text.

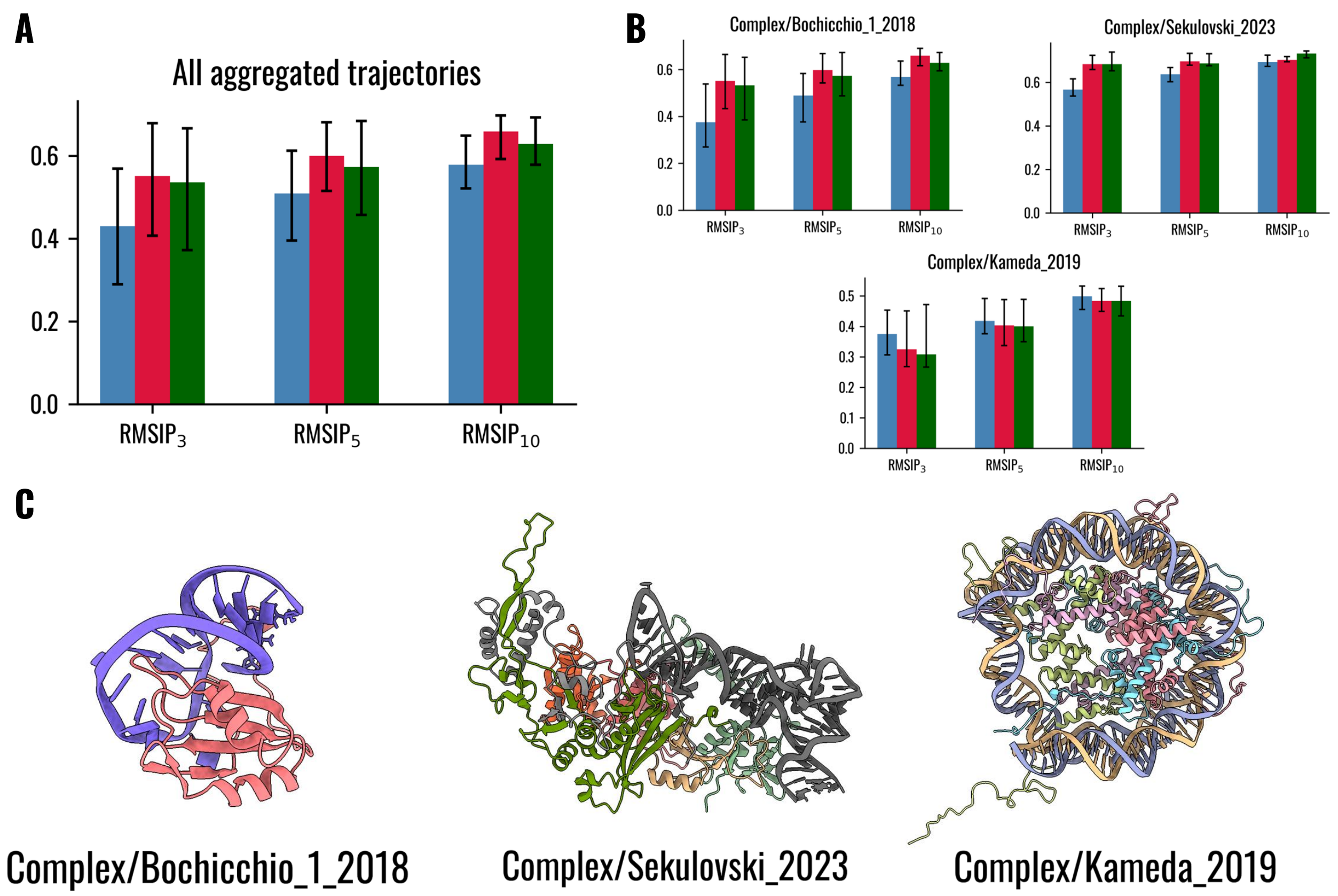

Supplementary Figure 13. Comparison between ENM (blue), edENM-PNA (red), and edProt (green) in terms of agreement with the conformational dynamics observed in the three test MD simulations for protein-nucleic acid systems. (A) Average RMSIP values ($RMSIP_3$, $RMSIP_5$, $RMSIP_{10}$) aggregated for the three testing MD simulations. Error bars refer to the first and third quantiles across the different MD trajectory windows. (B) Comparison with individual MD simulations from Bochicchio et al.[10], Sekulovski et al.[22], and Kameda et al.[17]. A graphical representation of each molecular system is shown in (C).

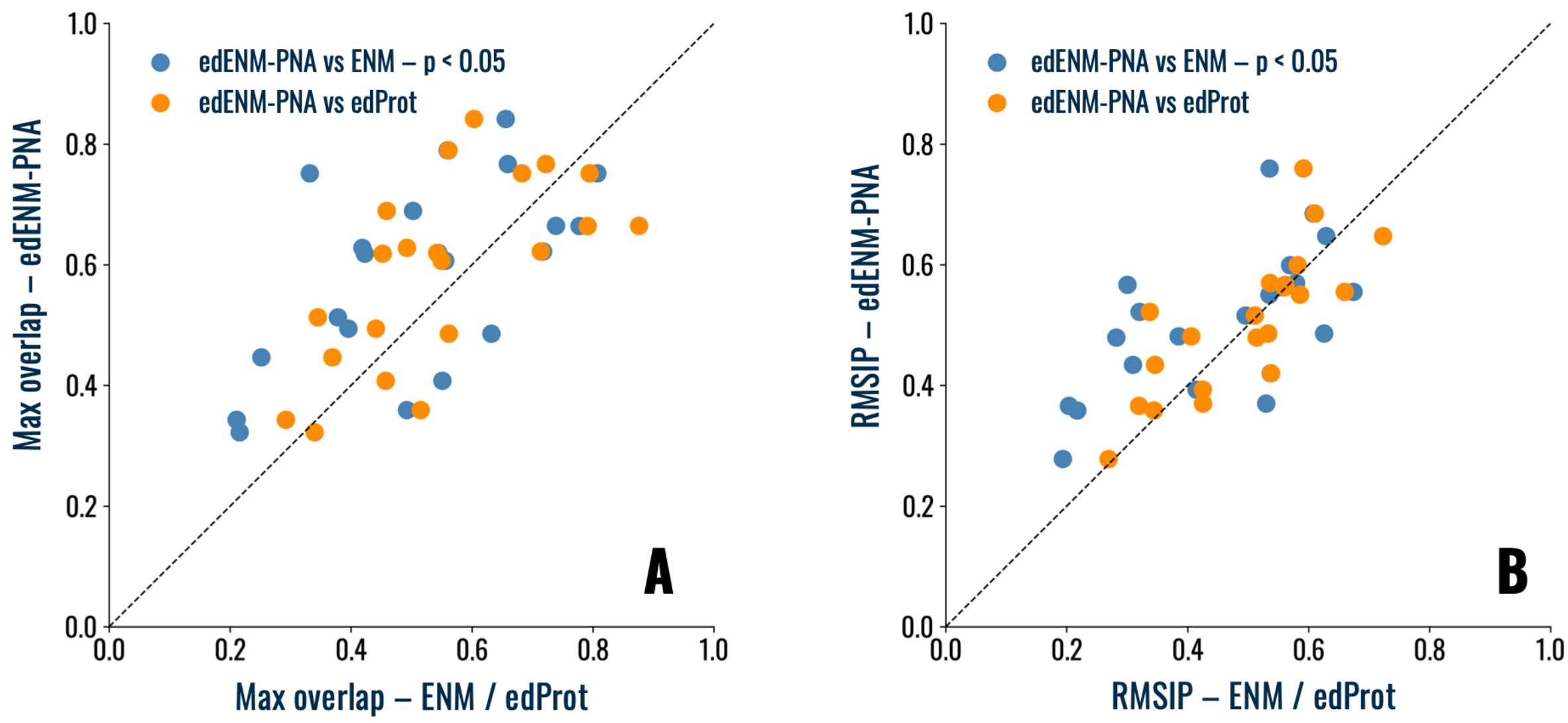

Supplementary Figure 14. Scatterplot showing the comparison between the edENM-PNA and ENM (blue points) or edProt (orange points) in terms of ability to capture experimental PCs from the set of NMR structures of protein-nucleic acid systems (Supplementary Table 3). (A) O_{max} for the set of first 3 NMs and experimental PCs. (B) RMSIP for the set of first 3 NMs and experimental PCs. *P*-values arising from one-sided Wilcoxon tests between O_{max} and RMSIP scores are reported in Supplementary Table 9.

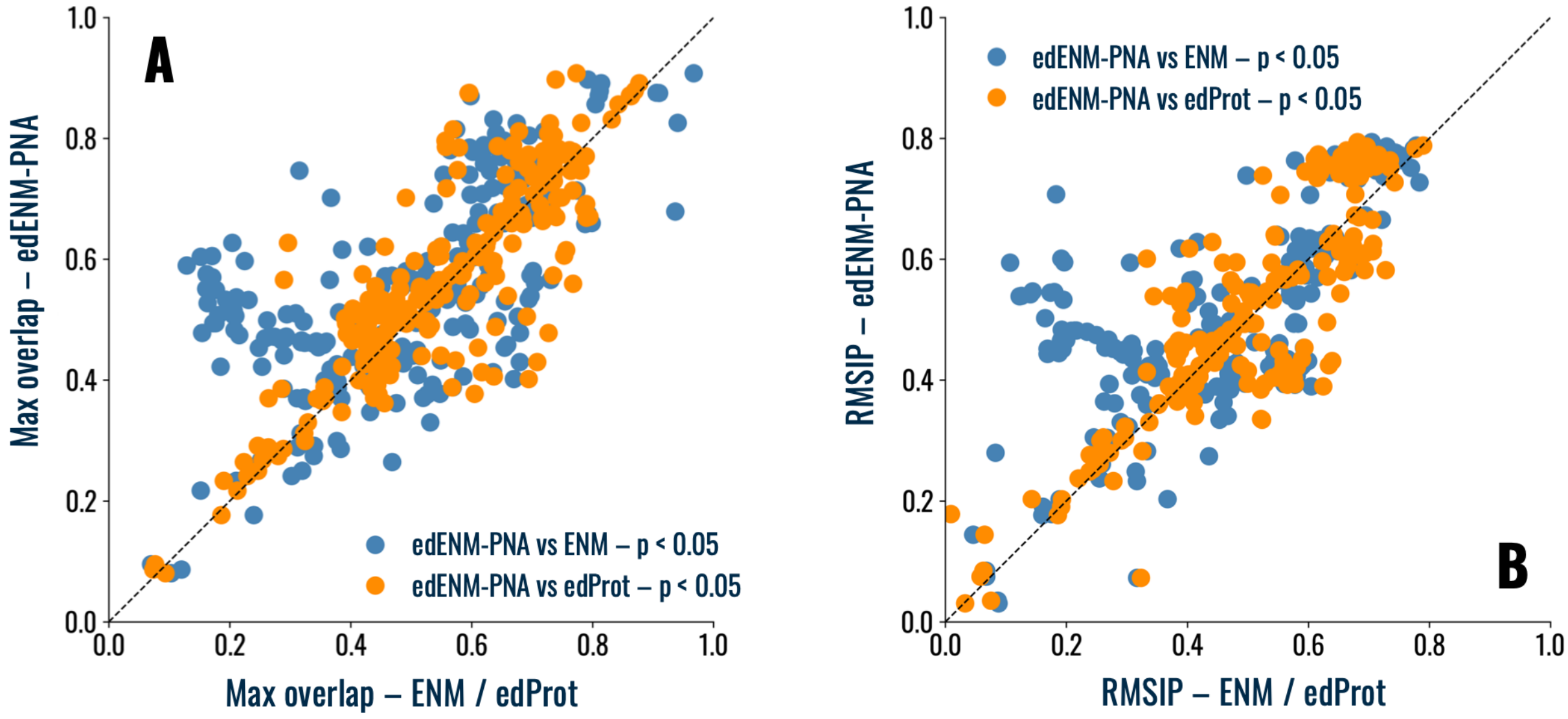

Supplementary Figure 15. Scatterplot showing the comparison between the edENM-PNA and ENM (blue points) or edProt (orange points) in terms of ability to capture experimental PCs from the set of protein-nucleic acid conformational ensembles at the level of individual PDB models (Supplementary Table 4). (A) O_{max} for the set of first 3 NMs and experimental PCs. (B) RMSIP for the set of first 3 NMs and experimental PCs. Each point corresponds to an individual PDB. *P*-values arising from one-sided Wilcoxon tests between O_{max} and RMSIP scores are reported in Supplementary Table 11.

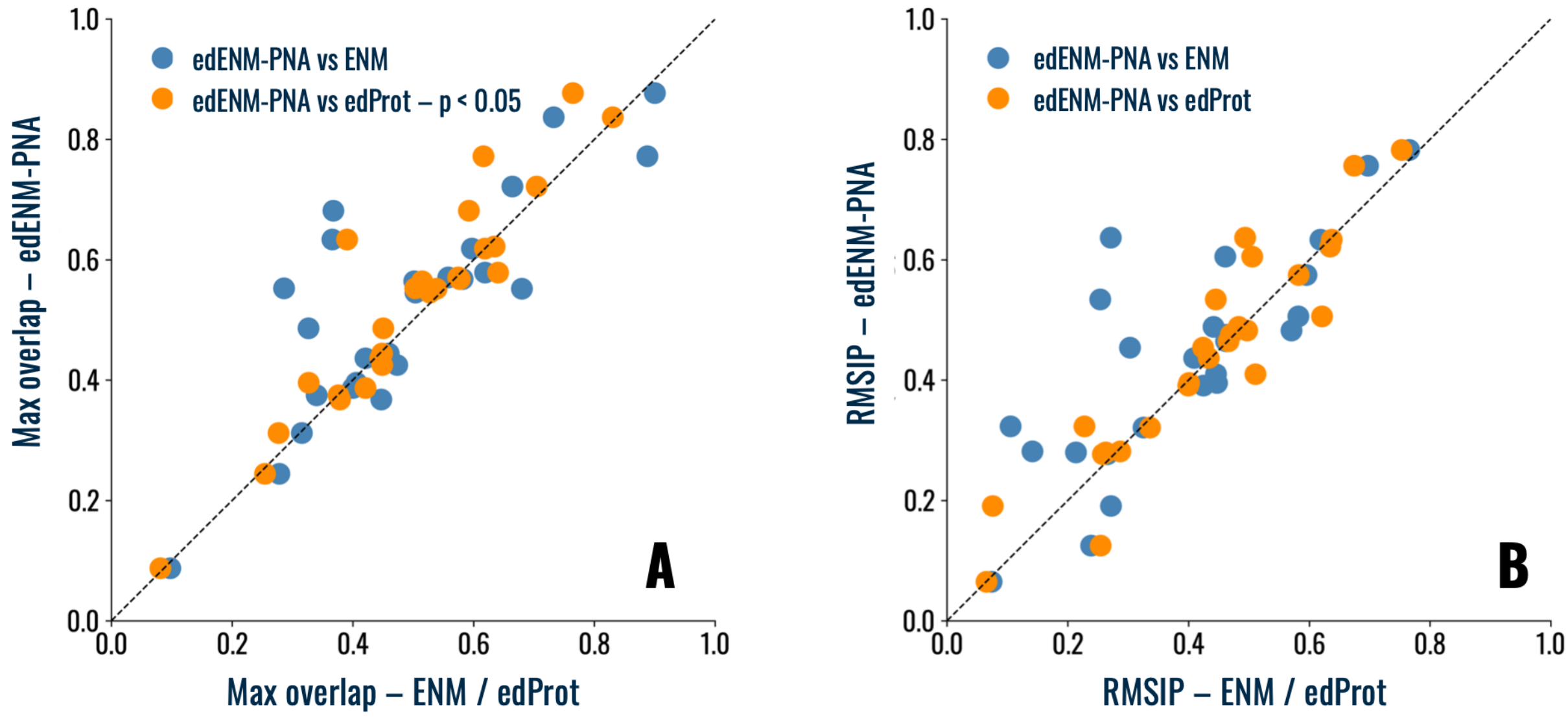

Supplementary Figure 16. Scatterplot showing the comparison between the edENM-PNA and ENM (blue points) or edProt (orange points) in terms of ability to capture experimental PCs from the set of protein-nucleic acid conformational ensembles, considering average similarity scores per ensemble. (A) O_{max} for the set of first 3 NMs and experimental PCs. (B) RMSIP for the set of first 3 NMs and experimental PCs. Each point corresponds to the average score for each individual ensemble. *P*-values arising from one-sided Wilcoxon tests between O_{max} and RMSIP scores are reported in Supplementary Table 12.

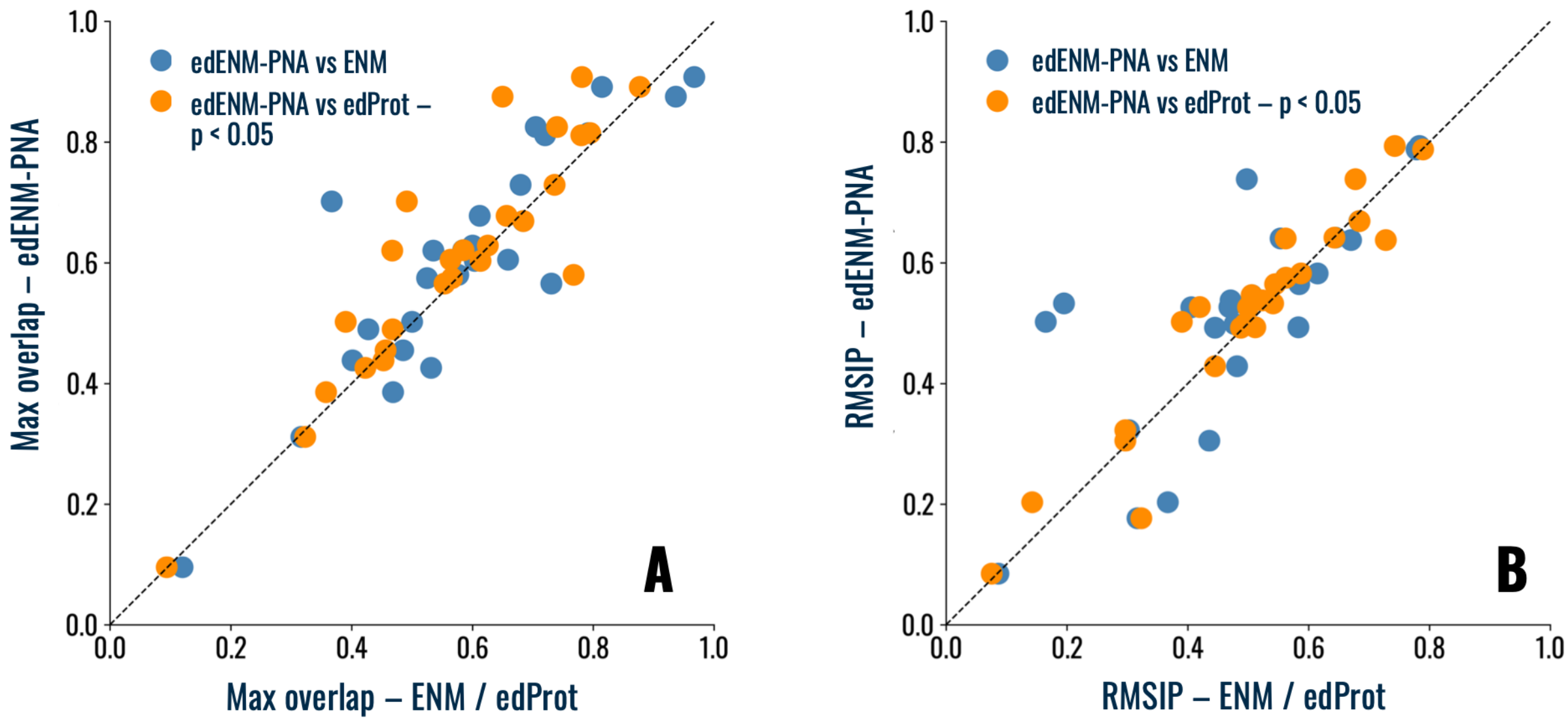

Supplementary Figure 17. Scatterplot showing the comparison between the edENM-PNA and ENM (blue points) or edProt (orange points) in terms of ability to capture experimental PCs from the set of protein-nucleic acid conformational ensembles, considering maximum similarity scores per ensemble. (A) O_{max} for the set of first 3 NMs and experimental PCs. (B) RMSIP for the set of first 3 NMs and experimental PCs. Each point corresponds to the maximum score for each individual ensemble. *P*-values arising from one-sided Wilcoxon tests between O_{max} and RMSIP scores are reported in Supplementary Table 13.

References

1. Vreede, J., Pérez de Alba Ortíz, A., Bolhuis, P. G. & Swenson, D. W. H. Atomistic insight into the kinetic pathways for Watson–Crick to Hoogsteen transitions in DNA. *Nucleic Acids Research* **47**, 11069–11076 (2019).

2. Vreede, J. & Swenson, D. W. H. Molecular dynamics simulations of DNA. (2017).

3. Lemkul, J. A. Same fold, different properties: polarizable molecular dynamics simulations of telomeric and TERRA G-quadruplexes. *Nucleic Acids Research* **48**, 561–575 (2020).

4. Polêto, M., Michel, H. M., Ratnasinghe, B., Salsbury, A. M. & Lemkul, J. A. Nucleic Acid Dynamics. https://doi.org/10.17605/OSF.IO/2PFJ8 (2017) doi:10.17605/OSF.IO/2PFJ8.

5. Kameda, T., Suzuki, M. M., Awazu, A. & Togashi, Y. Structural dynamics of DNA depending on methylation pattern. *Phys. Rev. E* **103**, 012404 (2021).

6. Kameda, T., Suzuki, M. M., Awazu, A. & Togashi, Y. Structural dynamics of DNA depending on methylation pattern: Simulation dataset. Zenodo https://doi.org/10.5281/ZENODO.3992686 (2020).

7. Pyne, A. L. B. *et al.* Base-pair resolution analysis of the effect of supercoiling on DNA flexibility and major groove recognition by triplex-forming oligonucleotides. *Nat Commun* **12**, 1053 (2021).

8. Pyne, A. L. B. *et al.* Base-pair resolution analysis of the effect of supercoiling on DNA flexibility and major groove recognition by triplex-forming oligonucleotides. (2021).

9. Salsbury, A. M., Michel, H. M. & Lemkul, J. A. Ion-Dependent Conformational Plasticity of Telomeric G-Hairpins and G-Quadruplexes. *ACS Omega* **7**, 23368–23379 (2022).

10. Bochicchio, A. *et al.* Molecular basis for the increased affinity of an RNA recognition motif with re-engineered specificity: A molecular dynamics and enhanced sampling simulations study. *PLoS Comput Biol* **14**, e1006642 (2018).

11. Bochicchio, A. *et al.* Molecular Basis For The Increased Affinity Of An Rna Recognition Motif With Re-Engineered Specificity: A Molecular Dynamics And Enhanced Sampling Simulations Study-Part 2. Zenodo https://doi.org/10.5281/ZENODO.1310053 (2018).

12. Bochicchio, A. *et al.* Molecular Basis For The Increased Affinity Of An Rna Recognition Motif With Re-Engineered Specificity: A Molecular Dynamics And Enhanced Sampling Simulations Study.-Part 8. Zenodo https://doi.org/10.5281/ZENODO.1465433 (2018).

13. Levintov, L. & Vashisth, H. Role of conformational heterogeneity in ligand recognition by viral RNA molecules. *Phys. Chem. Chem. Phys.* **23**, 11211–11223 (2021).

14. Levintov, L. & Vashisth, H. Dataset for: Role of conformational heterogeneity in ligand recognition by viral RNA molecules. Zenodo https://doi.org/10.5281/ZENODO.4521164 (2021).

15. De Bisschop, G. *et al.* Progress toward SHAPE Constrained Computational Prediction of Tertiary Interactions in RNA Structure. *ncRNA* **7**, 71 (2021).

16. De Bisschop, G. *et al.* Progress Toward SHAPE Constrained Computational Prediction of Tertiary Interactions in RNA Structure. Zenodo https://doi.org/10.5281/ZENODO.5642809 (2021).

17. Kameda, T., Awazu, A. & Togashi, Y. Histone Tail Dynamics in Partially Disassembled Nucleosomes During Chromatin Remodeling. *Front. Mol. Biosci.* **6**, 133 (2019).

18. Kameda, T., Awazu, A. & Togashi, Y. Histone tail dynamics in partially disassembled nucleosomes during chromatin remodeling: Simulation dataset. Zenodo https://doi.org/10.5281/ZENODO.2548539 (2019).

19. Baltrukevich, H. & Bartos, P. RNA-protein complexes and force field polarizability. *Front. Chem.* **11**, (2023).

20. Baltrukevich, H. & Bartos, P. Cas12j-RNA simulations in ff19SB+OL3, ff14SB+OL3, OPLS4 and AMOEBA force fields. Zenodo https://doi.org/10.5281/ZENODO.7694878 (2023).

21. Baltrukevich, H. & Bartos, P. RIG-I and RNA complex MD simulations in ff19SB+OL3, ff14SB+OL3, OPLS4 and AMOEBA force fields. Zenodo https://doi.org/10.5281/ZENODO.7695265 (2023).

22. Sekulovski, S., Sušac, L., Stelzl, L. S., Tampé, R. & Trowitzsch, S. Structural basis of substrate recognition by human tRNA splicing endonuclease TSEN. *Nat Struct Mol Biol* **30**, 834–840 (2023).

23. Trowitzsch, S. & Stelzl, L. Protein-RNA complex simulation. Zenodo https://doi.org/10.5281/ZENODO.6513519 (2022).

24. Riccardi, E., Van Mastbergen, E. C., Navarre, W. W. & Vreede, J. Predicting the mechanism and rate of H-NS binding to AT-rich DNA. *PLoS Comput Biol* **15**, e1006845 (2019).

25. Vreede, J. Predicting the mechanism and rate of H-NS binding to AT-rich DNA. (2019).

26. Pinamonti, G., Bottaro, S., Micheletti, C. & Bussi, G. Elastic network models for RNA: a comparative assessment with molecular dynamics and SHAPE experiments. *Nucleic Acids Res* **43**, 7260–7269 (2015).
